# A leader-repeat hairpin blocks extraneous CRISPR RNA production in diverse CRISPR-Cas13 systems

**DOI:** 10.1038/s44318-026-00769-1

**Published:** 2026-04-02

**Authors:** Angela Migur, Maximilian Feussner, Chunyu Liao, Omer S Alkhnbashi, Adrien Chauvier, Nils G Walter, Rolf Backofen, Zasha Weinberg, Chase L Beisel

**Affiliations:** 1 Helmholtz Institute for RNA-based Infection Research (HIRI), Helmholtz-Centre for Infection Research (HZI), Würzburg, Germany; 2 Botnar Institute of Immune Engineering, Basel, Switzerland; 3 Bioinformatics Group, Department of Computer Science and Interdisciplinary Centre for Bioinformatics, University of Leipzig, Leipzig, Germany; 4 Faculty of Life and Health Sciences, Shenzhen University of Advanced Technology, Shenzhen, China; 5 Shenzhen Institutes of Advanced Technology, Chinese Academy of Sciences, Shenzhen, China; 6 Center for Applied and Translational Genomics (CATG), Mohammed Bin Rashid University of Medicine and Health Sciences (MBRU), Dubai Healthcare City, Dubai, United Arab Emirates; 7 College of Medicine, Mohammed Bin Rashid University of Medicine and Health Sciences (MBRU), Dubai Healthcare City, Dubai, United Arab Emirates; 8 Single Molecule Analysis Group and Center for RNA Biomedicine, Department of Chemistry, University of Michigan, Ann Arbor, USA; 9 Universität Freiburg, Freiburg, Germany; Signalling Research Centres BIOSS and CIBSS, University of Freiburg, Freiburg, Germany; 10 Bioinformatics Group, Department of Computer Science, University of Freiburg, Freiburg, Germany; 11 Medical Faculty, University of Würzburg, Würzburg, Germany

**Keywords:** CRISPR-Cas, Cas13b, CRISPR RNA, RNA Processing, RNA Structure, ecrRNA, Microbiology, Virology & Host Pathogen Interaction, RNA Biology

## Abstract

CRISPR RNAs (crRNAs) guide recognition and targeting of intracellular invaders as part of adaptive immunity by CRISPR-Cas systems. crRNAs are transcribed from CRISPR arrays of conserved repeats interlaced with invader-derived spacers. While crRNA production is essential for immunity, its optimization for defense remains poorly understood. Here, we show that, in diverse RNA-targeting type VI CRISPR-Cas systems, the leader RNA encoded upstream of the CRISPR array prevents formation of an invader-independent extraneous crRNA (ecrRNA) by blocking processing of the first repeat. Using the VI-B2 system from *Porphyromonas gingivalis* as a model, we demonstrate that the leader RNA and first repeat form a conserved inhibitory hairpin that precludes binding and processing by the system’s Cas13b nuclease. Disrupting this hairpin enables ecrRNA production, which in turn can deplete invader-derived crRNAs and reduce Cas13b-mediated phage defense. Structure prediction indicates that these leader-repeat hairpins are widespread across diverse type VI subtypes, highlighting a conserved regulatory mechanism. Our findings reveal how a prevalent branch of CRISPR-Cas systems suppresses ecrRNA formation to promote RNA-guided immunity.

## Synopsis

RNA-targeted CRISPR-Cas13 systems generate crRNAs from CRISPR arrays, although a non-functional crRNA should be produced from the array’s first repeat that would interfere with the immune response. This work shows that a conserved hairpin formed between the leader and first repeat blocks processing, preventing extraneous crRNA formation and maintaining effective Cas13 immunity.

- The leader RNA inhibits processing of the first repeat, preventing formation of the invader-independent extraneous CRISPR RNA.
- In the type VI-B2 system from *Porphyromonas gingivalis*, the leader and first repeat form a hairpin that prevents binding and processing by the Cas13b nuclease.
- Disruption of the hairpin enables ecrRNA production and reduces Cas13b-mediated phage defense.
- Bioinformatic analyses reveal widespread conservation of leader–repeat hairpins across diverse type VI CRISPR-Cas13 subtypes.

## Abstract (web summary)

A conserved leader–repeat hairpin prevents processing of the first CRISPR repeat, suppressing extraneous crRNA production and preserving Cas13-mediated immunity.

## Graphical abstract

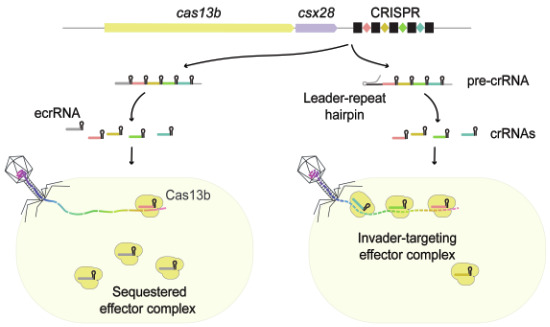

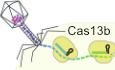

## Introduction

CRISPR (clustered regularly interspaced shortpalindromic repeats)-Cas (CRISPR-associated) systems in bacteria and archaea possess the unique ability to record past infections in the form of genomically-encoded and expandable CRISPR arrays (Barrangou and Marraffini, 2014). Each array stores fragments of foreign genetic material as DNA spacers flanked by conserved repeats. To leverage these records for immune defense, the CRISPR array is transcribed into a precursor CRISPR RNA (crRNA), or pre-crRNA (Charpentier et al, 2015). The pre-crRNA is then processed into individual crRNAs, with each crRNA encoding one of the stored spacers. The crRNA forms a ribonucleoprotein complex with the system’s effector nuclease, which then uses the processed spacer portion of the crRNA (called the guide sequence) to interrogate nucleic-acid sequences in the cell via specific base-pairing interactions (Charpentier et al, 2015). If a complementary nucleic-acid sequence flanked by an appropriate non-self motif is found (e.g., the protospacer-adjacent motif (PAM) in DNA targets and the protospacer-flanking sequence (PFS) in RNA targets), the nuclease is activated, leading to clearance of the targeted invader or shutdown of the infected cell (Gleditzsch et al, 2019).

Newly acquired spacers are added to one end of a CRISPR array (Barrangou et al, 2007; McGinn and Marraffini, 2016), providing a chronological record of past infections that can span hundreds of spacers (Pourcel et al, 2020). The repeat adjacent to the new spacer is copied in the process, maintaining the pattern of alternating repeats and spacers, with arrays beginning and ending with a repeat. One ramification is that each array contains an extra repeat that has the potential to be processed into a crRNA, yet it does not encode an invader-derived spacer. Instead, the guide sequence derives from outside of the array and thus is not inherently associated with immune defense. We termed these elements extraneous crRNAs (ecrRNAs) (Liao et al, 2019c).

**Figure 1:**
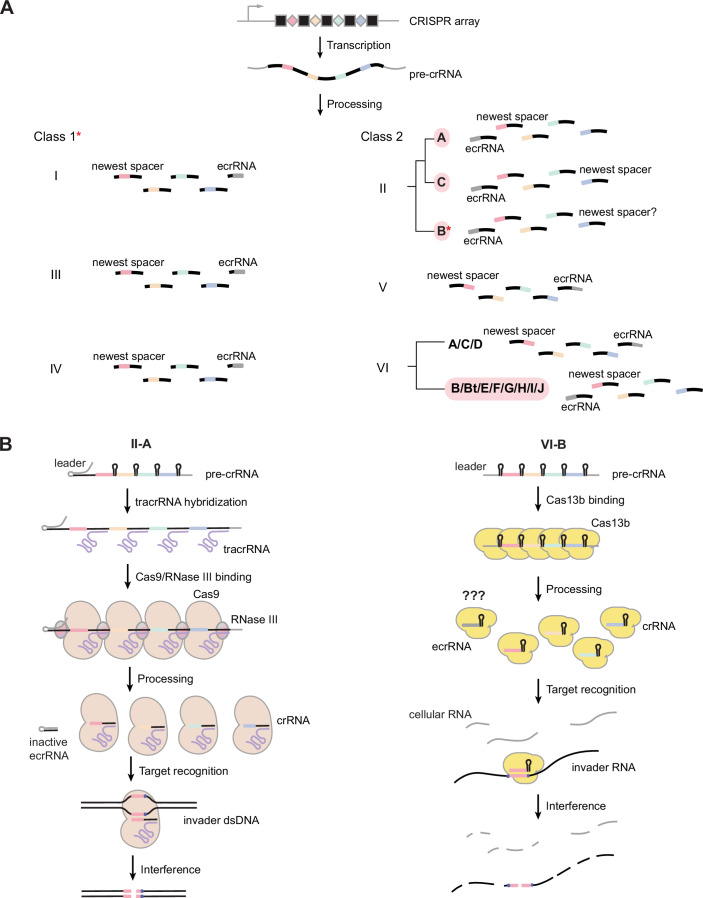
Extraneous crRNAs are sourced from the first repeat in multiple subtypes of type VI CRISPR-Cas systems. (**A**) The repeat associated with ecrRNA formation and spacer acquisition across CRISPR-Cas subtypes. In most cases, the ecrRNA would derive from the last repeat of the array opposite from the site of spacer acquisition and transcription initiation. Red asterisk: for Class I systems, the ecrRNA appears at the end since the 5′ end of each crRNA seeds effector complex formation, while the 3′ end is dispensable following processing. Types II-A and multiple type VI subtypes (including VI-B/Bt/E/F/G/H/I/J and ancestral type VI systems) are exceptions, where the ecrRNA would derive from the first repeat in the array associated with spacer acquisition. Red exclamation point: for II-B systems, the location of spacer acquisition and transcription remains to be determined. (**B**) Comparison of II-A and VI-B CRISPR-Cas systems. Left: crRNA biogenesis and interference in II-A systems. The tracrRNA hybridizes to each repeat of the pre-crRNA, and the resulting RNA duplex is cleaved by RNase III. The processed RNA duplex and Cas9 together form an effector complex that cleaves target dsDNA to clear an invader. Right: crRNA biogenesis and interference in VI-B systems. Cas13b recognizes a hairpin formed by the entire transcribed repeat, cleaving at the 3′ end of the bound repeat. Mature crRNAs guide Cas13b to target RNA. Upon target recognition, Cas13b cleaves RNAs non-specifically to induce cellular dormancy.

The location of the extra repeat, and with it the ecrRNA, depends on how the crRNA is configured (Fig. 1A). A guide-repeat configuration of the crRNA (with the guide upstream of its associated repeat) places the ecrRNA closest to transcription initiation when an upstream promoter drives transcription of the CRISPR array. In contrast, a repeat-guide configuration (with the repeat upstream of the guide) places the ecrRNA on the opposite end of the CRISPR array, farthest from transcription initiation. In the latter configuration, most common across system types and subtypes, the corresponding repeat is the oldest repeat. Accordingly, this repeat can accumulate mutations to prevent ecrRNA formation without impinging on the acquisition of new spacers, as we reported for V-A systems (Liao et al, 2019a, 2019c) and other CRISPR-Cas systems (Mitrofanov et al, 2025; Feussner et al, 2025). However, systems with the guide-repeat configuration cannot rely on disruptive mutations to prevent ecrRNA formation, as such mutations would also compromise the acquisition or function of new spacers.

**Figure EV1:**
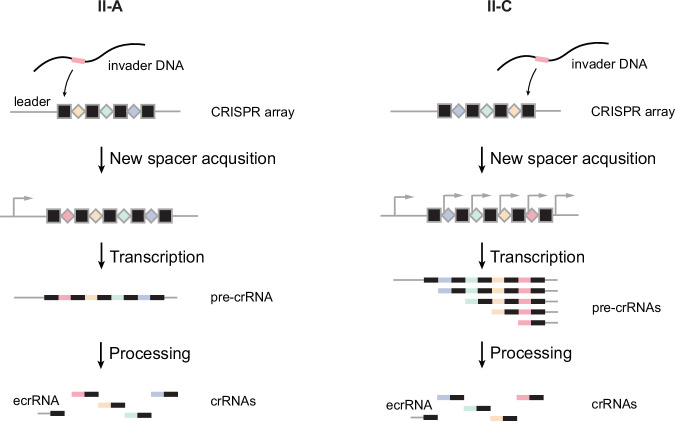
II-A and II-C CRISPR-Cas systems would both derive an extraneous crRNA from the first repeat adjacent to the newest spacer (II-A) or to the oldest spacer (II-C). The newest spacer is acquired through the first repeat in II-A systems or the last repeat in II-C systems. The CRISPR array is transcribed as a single pre-crRNA (II-A) or as multiple pre-crRNAs due to the repeats containing promoters (II-C). The ecrRNA would be formed at the beginning of the array from the first repeat adjacent to the newest spacer (II-A) or to the oldest spacer (II-C).

This realization led us to explore II-A CRISPR-Cas systems, which exhibit the guide-repeat configuration (Liao et al, 2022). We found that these systems frequently use the leader RNA encoded immediately upstream of the array to form a hairpin that interferes with hybridization of the trans-activating crRNA (tracrRNA). The resulting RNA duplex drives crRNA biogenesis (Deltcheva et al, 2011; Liao and Beisel, 2021). Apart from blocking ecrRNA formation, the hairpin accelerated maturation of the adjacent crRNA encoding the newest spacer in the array, possibly through direct interactions with the second repeat (Liao et al, 2022). Separately, we probed ecrRNA formation in II-C systems, which are an exception within CRISPR-Cas systems because each repeat encodes a promoter that drives the accumulation of crRNAs at the end of the array, with the last repeat serving as the site of spacer acquisition (Fig. EV1) (Dugar et al, 2013; Zhang et al, 2013; Hoikkala et al, 2021; Feussner et al, 2025). We showed that the II-C subtype also accumulates mutations in the first repeat that can disrupt ecrRNA formation, although they encode an upstream Rho-independent terminator or a hairpin structure that traps the ecrRNA spacer (Feussner et al, 2025). While these features are present across II-A and II-C systems, it remains unclear whether the leader’s role in optimizing crRNA production extends to other subtypes of CRISPR-Cas systems.

Here, we show that most subtypes of type VI CRISPR-Cas systems, RNA-targeting systems with the guide-repeat crRNA configuration but otherwise wholly distinct from II-A systems, utilize the leader RNA to block ecrRNA formation. Similar to II-A systems, the set of type VI systems forms a predicted hairpin with the first repeat, which prevents ecrRNA formation by blocking binding and subsequent processing by the Cas13 effector nuclease. In contrast to II-A systems (Fig. 1B), this hairpin does not facilitate spacer prioritization as part of immunity by a model VI-B2 system, although disrupting the hairpin can reduce the production of all crRNAs and impair anti-phage defense. These results reflect convergent evolution of the leader RNA to interfere with ecrRNA formation and suggest an evolutionary selection pressure to prevent ecrRNA formation for an optimal RNA-guided immune response.

## Results

### The leader and ecrRNA-forming repeat are predicted to form hairpins across multiple type VI subtypes

Given our finding that the leader RNA in II-A systems can block ecrRNA formation from the first repeat, we asked if other types or subtypes of CRISPR-Cas systems derive an ecrRNA from this repeat and thus may have evolved mechanisms to counteract ecrRNA formation. The vast majority of systems derive the ecrRNA from the oldest repeat typically located at the end of the array (Figs. 1A and EV1) (Mitrofanov et al, 2025; Feussner et al, 2025). In contrast, Type VI CRISPR-Cas systems within the VI-B and recently reported VI-Bt, VI-E, VI-F, VI-G, VI-H, VI-I, VI-J, and ancestral type VI subtypes utilize crRNAs that, like II-A systems, comprise a spacer-repeat crRNA configuration and derive the ecrRNA from the first repeat (Fig. 1A) (Hu et al, 2022; Yoon et al, 2024; Li et al, 2024). Unlike II-A systems, however, type VI systems utilize an RNA-targeting Cas13 nuclease that elicits collateral cleavage of non-specific RNAs (Fig. 1B). Moreover, these systems lack the tracrRNA, with Cas13 processing the repeats through recognition of an internal hairpin and cleavage at or near the 3′ end of each repeat. The repeats also form a large hairpin covering the canonical 30 nts, which would complicate the formation of a distinct hairpin with the leader RNA. Interestingly, the other type VI subtypes (i.e., VI-A/C/D) possess the repeat-guide crRNA configuration and derive the ecrRNA from the last repeat, suggesting distinct evolutionary paths to recognizing and utilizing crRNAs for immune defense.

**Figure 2:**
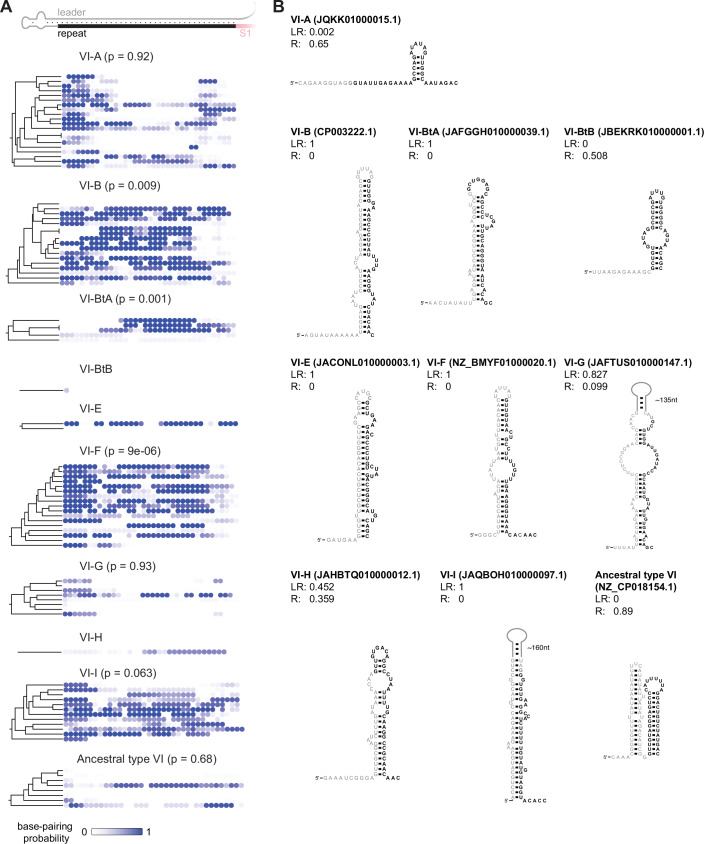
The transcribed leader and first repeat across VI-B CRISPR-Cas systems form a hairpin that competes with repeat folding. (**A**) Prediction of leader-repeat hairpins in VI-B/BtA/BtB/E/F/G/H/I and ancestral type VI systems. Similar predictions for VI-A systems serve as a negative control. Each dot represents a nucleotide of the first repeat and its probability to be base-paired with the leader. Aggregated *p* values represent the *p* values obtained for each system by comparing the probability that the leader-repeat hairpin versus 10,000 scrambled leaders disrupt all base pairs of the functional repeat and aggregated by Fisher’s method (see Methods). No aggregated *p* value is shown for systems with fewer than five non-redundant systems. We were unable to identify systems within the VI-J subtype. (**B**) Representative leader-repeat hairpins for each subtype; leader is gray, repeat is black. The probabilities of forming a leader-repeat hairpin (LR) or a repeat hairpin (R) are shown for each representative example.

We began by determining the extent to which the identified set of type VI subtypes encodes leader RNAs predicted to form hairpins with the first repeat. Scanning 180 nucleotides (nts) upstream of the first repeat, paralleling our prior interrogation of II-A systems (Liao et al, 2022), we identified examples within the VI-B/BtA/E/F/G/H/I subtype in which the first repeat preferentially forms a hairpin with the upstream leader rather than an internal hairpin (Fig. 2A,B; Dataset EV1). In contrast, we did not observe any examples for the VI-BtB subtype, possibly because only one example was available for this subtype. Analyzing the subtypes VI-B/BtA/F/G/I harboring at least five non-redundant systems for statistical power, we compared the extent of helix formation between the inferred leader and first repeat with the natural versus 1,000 scrambled leader sequences (Dataset EV2) (Liao et al, 2022). The leader-repeat hairpin appeared significantly more frequently than expected by chance for the VI-B (*p* = 0.009), VI-BtA (*p* = 0.001), and VI-F (*p* = 9 × 10^−6^) subtypes (Fig. 2A,B). For the VI-I subtype, the leader-repeat hairpin exhibited borderline statistical significance (*p* = 0.063), although the lack of statistical significance may be attributed in part to the low number of available non-redundant systems. Under half of VI-G systems contained a leader-repeat hairpin, explaining why leader-repeat hairpins did not appear more frequently than expected by chance across this subtype (*p* = 0.93). Interestingly, the ancestral type VI subtype did not contain any obvious leader-repeat hairpins and instead contained predicted hairpins within the guide portion of the ecrRNA, more closely paralleling that observed with II-C systems (Feussner et al, 2025). Importantly, VI-A systems, the most abundant subtype where the ecrRNA derives from the last repeat, show no evidence of a leader-repeat hairpin, with hairpins forming no more frequently than expected by chance (*p* = 0.92). A leader-repeat hairpin is thus predicted to form across the majority of Type VI subtypes in which the ecrRNA derives from the first repeat.

### The VI-B2 system from *Porphyromonas gingivalis* AJW4 forms a leader-repeat hairpin

**Figure 3:**
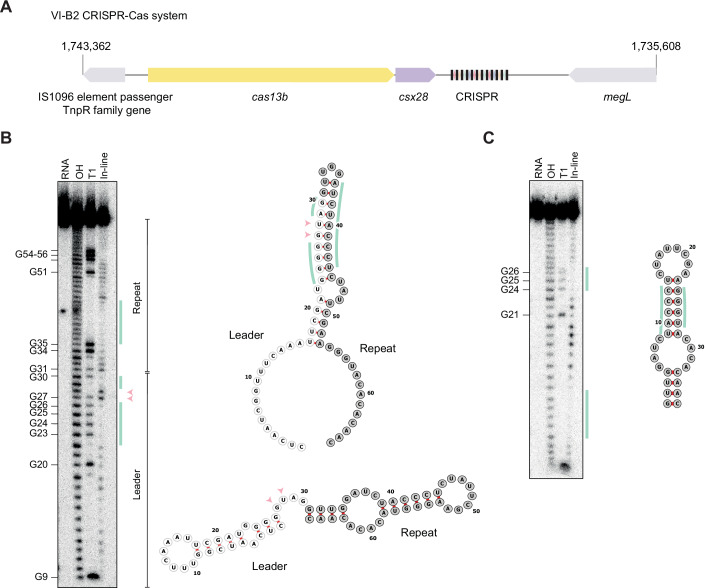
The VI-B2 CRISPR-Cas system from *Porphyromonas gingivalis* AJW4 forms a leader-repeat hairpin that competes with the formation of the internal repeat. (**A**) The VI-B2 CRISPR-Cas system from *Porphyromonas gingivalis* AJW4 (GenBank: CP011996.1). The representative VI-B system consists of *cas13b*, *csx28* and a CRISPR array with 11 spacers. (**B**) In vitro experimental determination of the structure associated with the leader-repeat RNA from the VI-B2 system in *P. gingivalis* AJW4. Left: in-line probing of the 5′ radiolabeled 66-nt RNA. RNA, untreated. OH, partial alkaline treatment. T1, RNase T1 treatment. Right: predicted minimal free-energy (MFE) structure of the leader-repeat without constraints (top) or constraining G27 or U28 to be single-stranded (bottom). The base-paired nucleotides are marked with green bars. The single-stranded nucleotides are marked with pink arrowheads. Gel images are representative of two independent experiments. (**C**) Experimental determination of the structure in vitro associated with the 36-nt repeat RNA from the VI-B2 system in *P. gingivalis* AJW4. See (**A**) for details. Gel images are representative of two independent experiments. Source data are available online for this figure.

**Figure EV2:**
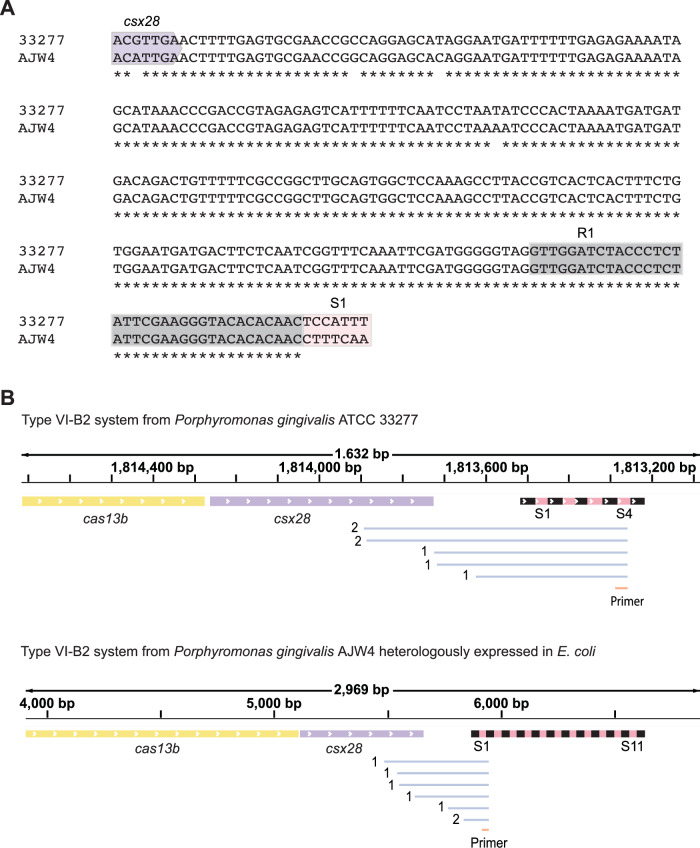
Transcription of the CRISPR array in the *P. gingivalis* VI-B2 system is initiated far upstream of the array. (**A**) Alignment of the first repeat and the sequence upstream of it in *P. gingivalis* ATCC 33277 and *P. gingivalis* AJW4. The first repeat and the upstream portion forming the leader-repeat stem-loop is identical between the two. (**B**) Alignment of the primary transcripts of the array (in blue) obtained through 5′ RACE to the Type VI-B system. The number of the sequenced primary transcripts with the same 5′ sequence is shown at the beginning of the transcript. The RNA subjected to 5′ RACE was extracted from *P. gingivalis* ATCC 33277 (NC_010729.1) (top) or from the *E. coli* strain heterologously expressing the VI-B system from *P. gingivalis* AJW4 on the plasmid pAM250 (bottom). The primer used for the PCR amplification of the transcripts converted into cDNA is in orange.

**Figure EV3:**
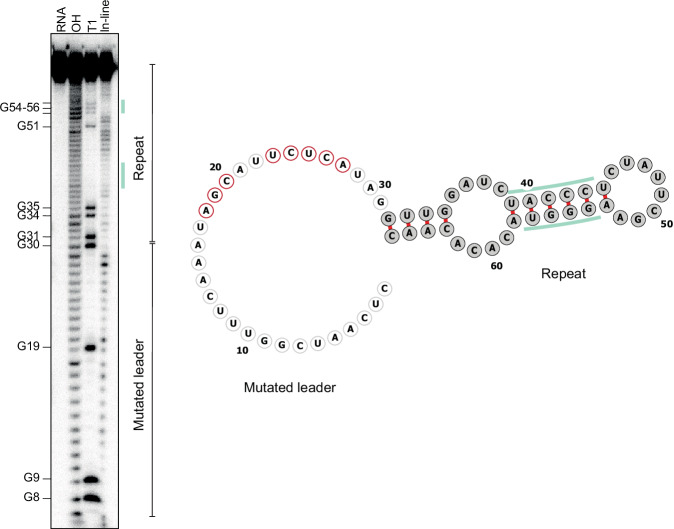
Mutating the leader disrupting the leader-repeat stem-loop restores the internal hairpin in the repeat. In-line probing of ~0.2 pmol P^32^-labeled mutated ecrRNA (mecrRNA). Spontaneous cleavage of single-stranded regions was analyzed on a 10% polyacrylamide gel with 7 M urea. Untreated RNA (lane RNA), partially alkali (lane OH) or RNase T1 (lane T1) digested RNAs served as ladders. The double-stranded nucleotides are marked with green vertical bars. The displayed gel is representative of duplicate independent experiments. The MFE structure is computationally predicted with RNAfold.

To further validate the predicted leader-repeat hairpins, we assessed the VI-B2 system from *Porphyromonas gingivalis* AJW4 as a representative example with extensive predicted base-pairing (Fig. 3A). This system encodes the effector nuclease Cas13b (abbreviated PgiCas13b), an accessory protein Csx28 that enhances Cas13b activity through triggered membrane destabilization (Smargon et al, 2017; VanderWal et al, 2023), and an 11-spacer array with repeat lengths of 36 bp and spacer lengths of 30 bp. Using 5′ RACE to map the transcriptional start site of the leader identified numerous transcript ends extending into the upstream *csx28* gene when heterologously expressing the system in *E. coli* or in the related native system residing in *P. gingivalis* ATCC 33277, indicating that the leader and CRISPR array are transcribed with the upstream *cas* genes (Fig. EV2). To resolve the secondary structure of the leader-repeat RNA, we performed in-line probing that reveals single-stranded regions of folded RNAs in vitro based on their propensity to undergo self-cleavage through nucleophilic attack of the phosphate backbone driven by the adjacent ribose hydroxyl group (Soukup and Breaker, 1999). In-line probing of the isolated leader-repeat confirmed preferential formation of this hairpin over the hairpin formed within the repeat (Fig. 3B,C). Accordingly, mutating the leader to disrupt formation of the leader-repeat hairpin resulted in folding of the repeat hairpin (Fig. EV3). Formation of this hairpin under active transcription was confirmed through an RNA polymerase pausing assay with the native or mutated leader, where the latter showed enhanced pausing after the repeat in line with repeat hairpin formation (Appendix Fig. S1). Together, these results show that the leader RNA and first repeat in the representative VI-B2 system form the predicted hairpin, which in turn competes with folding of the repeat hairpin.

### The leader-repeat hairpin prevents binding and processing by PgiCas13b

**Figure 4:**
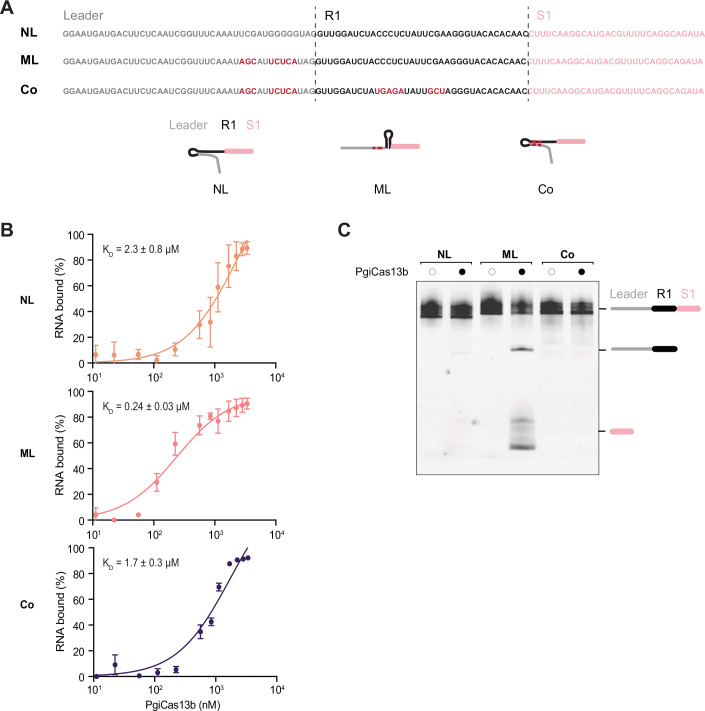
Binding and processing of the ecrRNA by Cas13b is inhibited by the leader-repeat hairpin in vitro. (**A**) 109 nt-long T7-transcribed single-spacer pre-crRNA of the type VI-B2 system from *P. gingivalis* AJW4 (NL) and the mutants (ML, Co). NL, native leader. ML, mutated leader designed to disrupt the leader-repeat hairpin. Co, mutated leader combined with a mutated first repeat to restore the leader-repeat hairpin. The transcript contains a 43 nt-long leader followed by a 36-nt-long repeat and 30 nt-long spacer 1 (S1). Top: sequences of the pre-crRNAs. Bottom: cartoon of the predicted structure adopted by NL, ML, and Co. (**B**) Electrophoretic mobility shift assay (EMSA) graphs showing the affinity of PgiCas13b to the 3′-labeled pre-crRNAs (NL, ML, Co). PgiCas13b non-specifically binds to RNAs in vitro: in order to reduce the non-specific binding to the RNA, the samples were supplemented with the yeast RNA. The experiment was performed in a buffer containing EDTA to inhibit the pre-crRNA processing by PgiCas13b. Error bars depict the standard deviation of triplicate independent measurements. The corresponding gels can be found in Appendix Fig. S2. The binding affinities for the PgiCas13b to NL and to ML are significantly different (*p* = 0.0495). Each circle and error bar represents the mean and standard deviation of three independent replicates. The *p* value comparing the binding affinities of NL and ML was calculated using an unpaired *t*-test with two tails. (**C**) In vitro processing assay of the pre-crRNAs (NL, ML, or Co) with the purified PgiCas13b. The RNA was separated on 6% polyacrylamide gel with 7 M urea, stained with SYBR Green II. Source data are available online for this figure.

We next explored the direct consequences of the leader-repeat hairpin on ecrRNA formation, a process that requires Cas13b to first bind and then process the transcribed repeat (Smargon et al, 2017). For these experiments, we utilized the native and mutated leader as well as a version in which the repeat was mutated to reform the leader-repeat hairpin with the mutated leader (Fig. 4A). Notably, this mutant no longer forms the canonical repeat hairpin recognized by Cas13b. We first evaluated in vitro binding of purified PgiCas13b to the three leader-repeat variants using an electrophoretic mobility shift assay (EMSA). We created 109-nt pre-crRNAs spanning the leader through the first spacer present in the type VI-B2 system from *P. gingivalis* AJW4. EMSAs were performed in a binding buffer lacking Mg^2+^ and supplemented with EDTA to inhibit pre-crRNA processing by PgiCas13b (Fig. 4B). PgiCas13b weakly bound the native leader-repeat (*K*_d_ ≈ 2.3 µM), while mutating the leader to disrupt leader-repeat formation led to tighter binding (*K*_d_ ≈ 0.24 µM) (*p* = 0.0495). However, further mutating the repeat to restore the leader-repeat hairpin and concomitantly block formation of the repeat hairpin led to similarly weak binding (*K*_d_ ≈1.7 µM) compared to the native leader-repeat (*p* = 0.85). The transcribed leader-repeat hairpin thus blocks binding of the repeat by PgiCas13b.

The inhibited binding of the transcribed leader-repeat by PgiCas13b would be expected to further inhibit processing to a mature ecrRNA. To test this possibility directly, we performed an in vitro cleavage assay with the purified PgiCas13b and the 109-nt pre-crRNAs used in the binding assay (Fig. 4B) in a cleavage buffer supplemented with 0.5 mM MgCl_2_ (Fig. 4C). Consistent with our expectations, the native leader-repeat yielded minimal processing by PgiCas13b, whereas mutating the leader allowed processing. Introducing compensatory mutations in the repeat resulted in undetectable processing. Together, our data demonstrate that the leader-repeat hairpin blocks binding and subsequent processing of the ecrRNA.

### The leader-repeat hairpin prevents ecrRNA formation and subsequent targeting in *E. coli*

**Figure 5:**
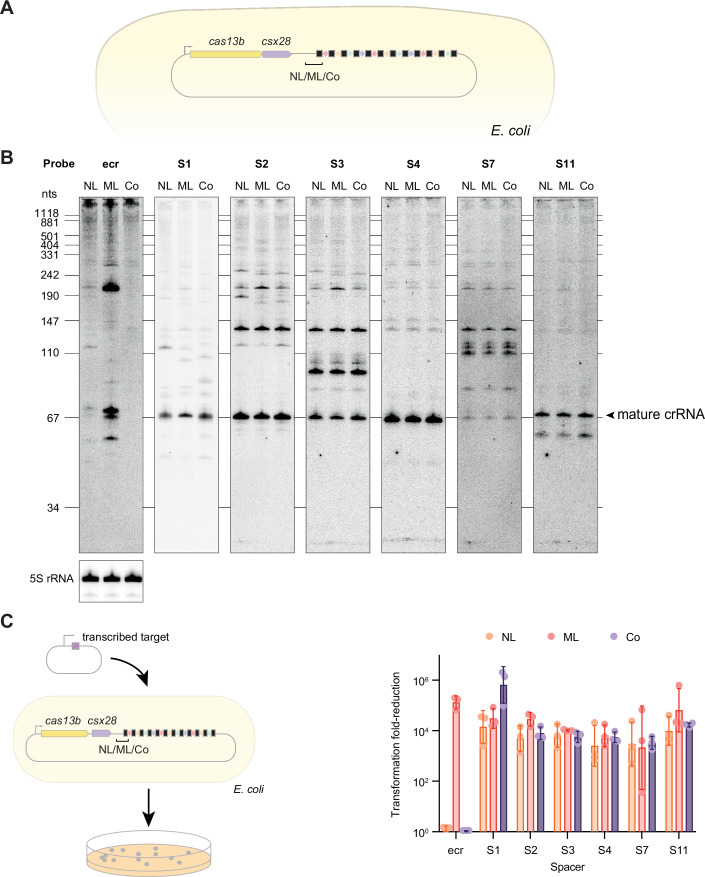
Disruption of the VI-B leader-repeat hairpin activates plasmid interference through the ecrRNA. (**A**) *E. coli* strains heterologously expressing the VI-B system from *P. gingivalis* AJW4 with the native leader (NL), mutated leader (ML), or mutated leader and repeat (Co). (**B**) Northern blotting analysis of the total RNA extracted from the *E. coli* strains heterologously expressing the VI-B system from *P. gingivalis* AJW4 (NL) or its mutations (ML, Co). RNA was separated on an 8% polyacrylamide gel with 7 M urea. ^32^P-labeled probes to the leader (ecr), spacers 1, 2, 3, 4, 7, and 11 were hybridized. To control the RNA loading, hybridization of the probe to 5S rRNA was performed. Results are representative of three independent experiments. (**C**) Plasmid interference assay schematic and results. The plasmids expressing targets corresponding to ecrRNA (ecr), spacers 1, 2, 3, 4, 7, or 11 of the array or no-target control conferring ampicillin resistance were co-transformed with a kanamycin-resistance plasmid encoding the VI-B2 system from *P. gingivalis* AJW4 (NL) or its mutants (ML, Co). The transformation was plated on double antibiotic selection plates. Each dot represents the measurement from one independent biological replicate, while the bars and error bars represent the geometric mean and geometric standard deviation of the three independent biological replicates. Source data are available online for this figure.

Building on these in vitro findings, we assessed the impact of the leader-repeat hairpin using the heterologous expression system in *E. coli* (Fig. 5A). To draw direct comparisons, we introduced the mutations to the leader as well as the double mutation of both the leader and first repeat (Fig. 4A). Consistent with reduced binding and processing by PgiCas13b in vitro, northern blotting analysis revealed that the mature ecrRNA as well as a longer version were detectable only with the mutated leader (Fig. 5B). The only consistent products were multi-kilobase transcripts likely encoding the entire pre-crRNA as well as the upstream *cas* genes. The lack of detectable ecrRNAs could be attributable to the lack of binding by PgiCas13b that would otherwise protect the ecrRNA from cellular RNases.

Using a plasmid interference assay, we next assessed the ability of the ecrRNA to drive the RNA-triggered activation of PgiCas13b and, subsequently, Csx28 (Fig. 5C). As part of the assay, a plasmid expresses an ecrRNA target that is capable of inducing cellular dormancy and a drop in colony counts. Paralleling the appearance of the mature ecrRNA by northern blotting analysis, the mutated leader led to an ~10^5^-fold reduction in colony counts compared to a non-targeting control. In contrast, the native leader-repeat as well as the double-mutated leader and repeat did not yield any measurable reduction. The lack of targeting by the native ecrRNA was not due to its particular location at the front of the array, as replacing individual spacers with the ecrRNA-matching spacer in the native array led to at most a 24-fold reduction in colony counts (Appendix Fig. S3). These results show that the leader-repeat hairpin interferes with the formation of a functional ecrRNA that otherwise primes PgiCas13b for RNA-guided activation.

### ecrRNA formation can reduce Cas13b-mediated phage defense

While the leader-repeat prevented the ecrRNA from being formed and directing activation of PgiCas13b, we asked how ecrRNA formation affects production and targeting activity of the other crRNAs encoded within the CRISPR array. Returning to northern blotting analysis, we probed crRNAs derived from six spacers (S1, S2, S3, S4, S7, and S11) across the CRISPR array. For all probed spacers, disrupting the leader-repeat hairpin had little to no impact on the abundance of the mature crRNAs (Fig. 5B). Similarly, performing the plasmid interference assay on plasmids expressing spacer targets resulted in similar colony reductions (Fig. 5C). At most, double-mutating the leader and first repeat resulted in a measurable yet insignificant enhancement of colony reduction through the first spacer.

**Figure EV4:**
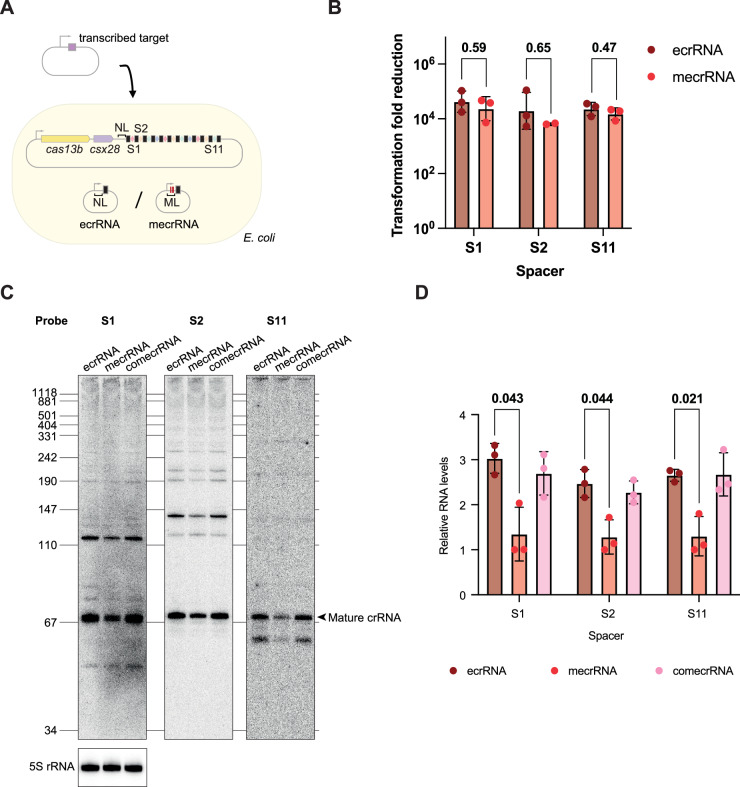
Expression of the ecrRNA with the disrupted leader-repeat stem-loop in *trans* decreases crRNA levels from the Type VI-B array. (**A**) The *E. coli* strain heterologously expressing the VI-B system from *P. gingivalis* AJW4 was co-transformed with the plasmids expressing a native ecrRNA (ecrRNA), the ecrRNA with the mutation in the leader (mecrRNA), or the ecrRNA with the mutations in the leader and repeat (comecrRNA). For the plasmid interference assay, an ampicillin-resistance plasmid expressing targets corresponding to spacers 1, 2, or 11 of the array, or a no-target control, was co-transformed with a kanamycin-resistance plasmid encoding the VI-B2 system from *P. gingivalis* AJW4 and a chloramphenicol-resistant plasmid expressing either ecrRNA or mecrRNA. The transformation was plated on triple-antibiotic selection plates. (**B**) Plasmid interference by Cas13b when expressing the ecrRNA in *trans*. The plasmid expressed a transcript targeted by spacer 1, 2, or 11, while the ecrRNA or mecrRNA was expressed in *trans*. The *p* values were calculated using a paired *t*- test with a two-tailed *p* value. The experiment was performed in two or three biological replicates. Each dot represents an independent biological replicate, while the bars and error bars represent the geometric mean and geometric standard deviation. (**C**) Northern blotting analysis of the total RNA extracted from the *E. coli* strains heterologously co-expressing the VI-B system from *P. gingivalis* AJW4 together with ecrRNA, mecrRNA, or comecrRNA. RNA was separated on 8% polyacrylamide gel with 7 M urea; ^32^P-labeled probes to the spacers 1, 2, and 11 were hybridized. To control the RNA loading, hybridization of the probe to 5S rRNA was performed. One representative replicate out of three is shown. (**D**) Quantification of the mature crRNAs abundance levels. The bands running at the size of mature crRNAs (at 66 nts) were used to measure intensities. 5S rRNA was used to normalize the intensities. Error bars depict the standard deviation of biological triplicates. Expression of mecrRNA significantly dropped the crRNAs transcript levels compared to the control strain expressing ecrRNA for spacers 1 (2.2-fold), 2 (1.9-fold), and 11 (2.2-fold). The *p* values were calculated using a paired *t*-test with two tails. Each dot represents an independent biological replicate, while the bars and error bars represent the mean and standard deviation.

Recent work showed that phage-encoded crRNAs, termed RNA anti-CRISPRs or Racrs, can inhibit CRISPR-based immunity in *trans* by titrating away available Cas nucleases (Camara-Wilpert et al, 2023). As the ecrRNA would be one of the most abundant crRNAs, as the closest to the transcriptional start site (Elmore et al, 2013; Carte et al, 2014; Crawley et al, 2018; Deltcheva et al, 2011), we asked whether expressing a functional ecrRNA in *trans* could affect Cas13b-mediated defense through our heterologous expression system in *E. coli*. When driving plasmid interference through different spacers, there was no significant difference in expressing the functional ecrRNA (i.e., mutated leader-repeat) compared to a non-functional ecrRNA (i.e., native leader-repeat) (Fig. EV4A,B). However, there was a small but significant reduction in all detected mature crRNAs (from S1, S2, S11) across the CRISPR array (Fig. EV4C,D), suggesting some effect of functional ecrRNA expression in *trans*.

**Figure EV5:**
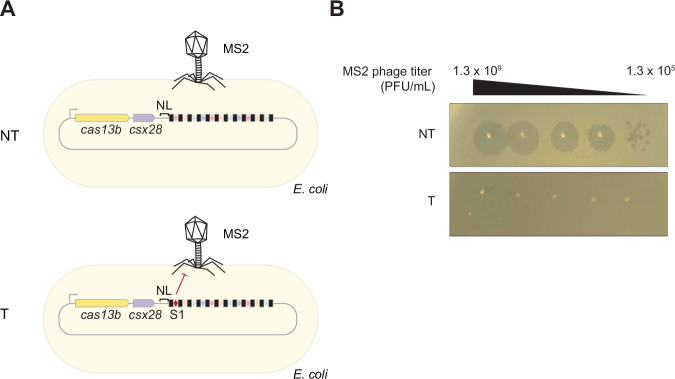
The CRISPR-Cas13b system confers efficient protection against the MS2 phage. (**A**) *E. coli* strains heterologously expressing the type VI-B CRISPR-Cas system from *P. gingivalis* AJW4, carrying either the native array (NT) or an array in which spacer 1 was replaced with an MS2-targeting spacer (T), were challenged with MS2 phage. (**B**) Plaque formation following infection with the lytic MS2 phage. A spacer targeting MS2 protected the cells from infection, resulting in the absence of visible plaques. Shown are regions of agar plates overlaid with soft agar containing *E. coli* strains, onto which serial dilutions of MS2 phage were spotted. The data were representative of three independent experiments.

**Figure 6:**
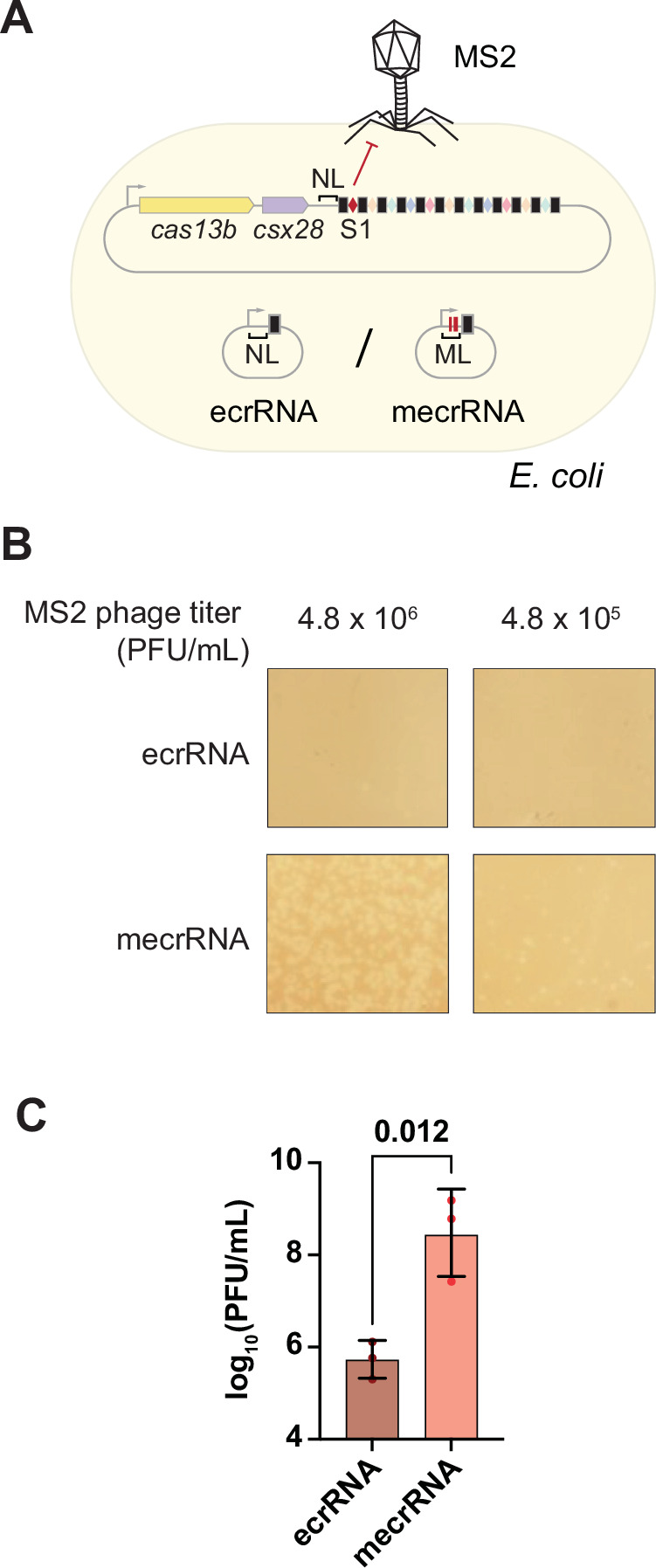
The ecrRNA with a disrupted leader-repeat hairpin can sequester Cas13b and inhibit protection against a phage when overexpressed in *trans*. (**A**) The *E. coli* strain heterologously expressing the VI-B system from *P. gingivalis* AJW4 with the spacer 1 replaced by the one targeting the MS2 phage was co-transformed with the plasmids expressing a native ecrRNA, or ecrRNA with the mutation in the leader (mecrRNA). (**B**) Plaque formation following infection with the lytic MS2 phage. Shown are zoomed-in regions of the agar plate overlaid with soft-agar mixed with *E. coli* and the MS2 phage. Results are representative of three independent experiments. (**C**) Quantification of the PFU/mL of the MS2 phage. Each dot represents the measurement from one independent biological replicate, while the bars and error bars represent the geometric mean and geometric standard deviation of the three independent biological replicates. The *p* value was calculated using a paired *t*-test with two tails based on log values. Source data are available online for this figure.

Building on this finding, we assessed the impact of ecrRNA expression on anti-phage defense. Here, we replaced spacer 1 in the native array with an MS2-targeting spacer, which conferred protection against MS2 infection in *E. coli* (Fig. EV5). We also placed the ecrRNA on a higher-copy plasmid to drive a potentially stronger inhibitory effect. In line with this expectation, overexpression of the functional ecrRNA resulted in an ~1000-fold increase in plaque formation compared to the non-functional ecrRNA (Fig. 6A–C). These results indicate that the processed and functional ecrRNA can compete with crRNAs for the available pool of Cas13b, presumably leading to an evolutionary pressure to prevent ecrRNA production.

## Discussion

**Figure 7:**
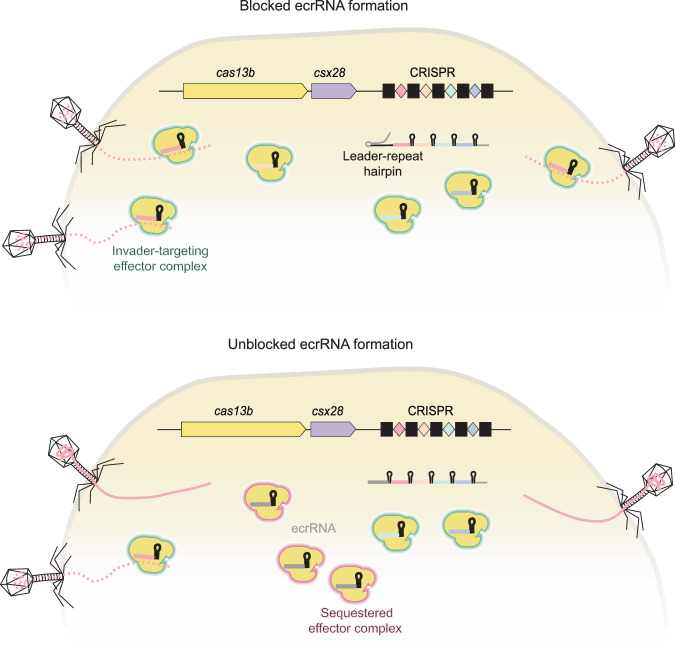
Proposed model for ecrRNA inhibition and its impact on immune optimization across type VI CRISPR-Cas systems. In type VI-B systems or other type VI subtypes with the guide-repeat crRNA configuration, expression of the ecrRNA is blocked by a hairpin structure formed between the leader and the first repeat of the pre-crRNA. Disruption of this leader-repeat hairpin results in ecrRNA expression, which sequesters Cas13b and inactivates the effector complex, leading to a depletion of active effector complexes available to combat phage infection.

Our findings reveal a prevalent mechanism across multiple type VI CRISPR-Cas systems in which the leader RNA forms a hairpin with the first repeat, blocking production of the ecrRNA. This hairpin disrupts the secondary structure of the first repeat within the pre-crRNA, preventing its recognition and processing by Cas13 nucleases. Based on our proposed model (Fig. 7), disruption of this structure allows ecrRNA production, which in turn sequesters Cas13 effector nucleases and reduces the availability of active effector complexes, potentially weakening the immune response.

This mechanism mirrors strategies recently reported in type II systems despite their distinct nucleases and use of a tracrRNA (Liao et al, 2022). Compared to II-A systems, where the hairpin both blocks ecrRNA formation and promotes maturation of the first crRNA (Liao et al, 2022), the leader-repeat hairpin in the VI-B2 system from *P. gingivalis* appears to solely prevent ecrRNA formation by obstructing Cas13b binding and processing without directly affecting the first crRNA. This underscores that, although structurally similar, leader-repeat interactions can serve distinct functional roles that depend on the CRISPR-Cas system. II-C systems also possess mechanisms that reduce ecrRNA formation, but act via alternative strategies such as internal promoters, structural elements upstream of the first repeat, or mutations in the repeat (Feussner et al, 2025), further illustrating the diversity of solutions evolved to address this shared challenge. The mechanism by which some subtypes, like II-B, transcribe and regulate their arrays remains unclear, where future work could clarify whether they too utilize leader-based strategies or distinct mechanisms to counter ecrRNA formation. Together, these observations point to a striking case of convergent evolution, where different CRISPR-Cas systems have independently developed mechanisms to address the shared constraint imposed by the guide-repeat configuration of crRNAs.

Interestingly, we do not observe a prominent leader-repeat hairpin in the recently identified VI-BtB, ancestral type VI and over half of VI-G systems (Fig. 2A,B). The observed guide hairpin in the ancestral type VI systems suggests that these CRISPR-Cas subtypes employ alternative ecrRNA inhibition strategies. Further investigation may reveal alternative regulatory mechanisms that compensate for the absence of the hairpin structure, offering further insights into the diversity of ecrRNA blocking strategies. In addition, the possibility remains that functional ecrRNAs have been co-opted for other purposes since they would remain fixed even as the CRISPR array expands or condenses.

More broadly, the diverse mechanisms that disrupt ecrRNA formation suggest a general evolutionary pressure across CRISPR-Cas systems to prevent the production of non-functional or potentially interfering RNAs. The importance of suppressing their expression is further underscored by the observation that some phages exploit crRNA-mimicking RNAs, known as Racrs (Camara-Wilpert et al, 2023). Racrs are encoded by phages that sequentially or only structurally resemble the repeats of crRNAs when expressed (Hayes et al, 2025). Racrs inactivate CRISPR effector complexes by sequestering the effector nucleases, effectively neutralizing host defense. Similarly, if ecrRNAs are capable of sequestering effector nucleases, they would functionally resemble Racrs. It is therefore unsurprising that CRISPR-Cas systems have evolved mechanisms to actively suppress ecrRNA expression.

Expressing the processed and mature functional ecrRNA from the VI-B2 system in *trans* indicated sequestering of available Cas13b nuclease molecules that reduced Cas13b-mediated phage defense by ~1000-fold (Fig. 6A–C). This reduction required overexpression of the functional ecrRNA in *trans* that would likely be less pronounced in a native system in which only a single ecrRNA is expressed at the beginning of the array. However, crRNAs from the beginning of an array are the most highly expressed (Elmore et al, 2013; Carte et al, 2014; Crawley et al, 2018; Deltcheva et al, 2011), which could introduce sufficient selective pressure to drive silencing of the ecrRNA through formation of the leader-repeat stem-loop. The selective pressure could be enhanced by longer CRISPR arrays or in the presence of Acr-expressing phages (Workman et al, 2021; Workman et al, 2024), in which immunity becomes sensitive to perturbations in the levels of individual crRNAs. It also remains possible that the ecrRNA serves a regulatory function paralleling scaRNAs and CRISPR-encoded antitoxin RNAs (Li et al, 2021; Sampson et al, 2013). Future work could therefore focus on understanding the widespread mechanisms to block ecrRNA formation on CRISPR-Cas immunity in native contexts.

## Methods

**Table:** Reagents and tools table

Reagent/resource	Reference or source	Identifier or catalog number
**Experimental models**		
*E. coli* RL73 (BW25113 ΔCRISPR ΔLacOperon)	Leenay et al, 2016	
*E. coli* TOP10	Invitrogen	C404010
*Porphyromonas gingivalis* ATCC 33277	ATCC	ATCC 33277
NEB® Turbo Competent *E. coli*	New England Biolabs	C2984H
Escherichia phage MS2 DSM 13767	DSMZ	DSM 13767
**Recombinant DNA**		
Plasmids	This study	Table EV1
**Oligonucleotides and other sequence-based reagents**		
Primers/DNA oligonucleotides	This study	Table EV2
RNAs	This study	Table EV3
**Chemicals, enzymes and other reagents**		
Chemicals, Enzymes and other reagents	This study	Table EV4
**Software**		
Prism version 10	GraphPad	
ImageQuant™ TL	Cytiva	

### Related materials

Primers, plasmids, and RNA sequences used in this work are listed in Tables EV1, EV2, and EV3. Chemicals, enzymes and other reagents are listed in Table EV4.

### Computational sequence and structure analysis

The full list of identified leader-repeat sequences is contained in Dataset EV1. To provide a meaningful statistical answer to the question of whether a leader-repeat hairpin is present in type VI-B, 113 VI-B systems were divided into independent training and test data sets. Sequences within the training and test sets had a maximum sequence identity of 70%, and between the sets a maximum sequence identity of 50% to ensure sufficient independence of samples for statistical testing. By sampling the Boltzmann distribution 1000 times using the ViennaRNA library, the probability that all base pairs of the functional repeat are opened by the leader-repeat interaction was calculated. The calculated probabilities were compared to 1000 sequences derived from the shuffle leaders (preserving dinucleotide frequency) to obtain an empirical *p* value. Empirical *p* values obtained from the test set (excluding sequences used in training) were aggregated into a single *p* value using Fisher’s method. For type VI-A, VI-BtA, VI-F, VI-G, and VI-I systems, no training/test data split was applied; instead, sequences were filtered using a 70% identity threshold, and empirical *p* values were calculated directly on these filtered sets. For subtypes VI-BtB, VI-E, and VI-H, fewer than five systems remained after applying the identity filter, and thus these subtypes were excluded from statistical analysis. Instead, they were manually inspected for potential leader-repeat hairpin interactions. The list of leader-repeat sequences subjected to the statistical analyses and displayed in Fig. 2A are contained in Dataset EV2.

### Primers, plasmids, and growth conditions

All primers and gBlocks were ordered from Integrated DNA Technologies. Q5 site-directed mutagenesis kit (New England Biolabs) was used for small insertions and nucleotide substitutions. NEBuilder HiFi DNA Assembly Master Mix (New England Biolabs) was used to clone long dsDNA fragments into a plasmid.

For the construction of the plasmid expressing the type VI-B from *Porphyromonas gingivalis* AJW4 (pAM0250), gBlocks were used for cloning of the Cas13b and Csx28; the array was assembled using the CRATES method (Liao et al, 2019b).

*E. coli* cells were grown at 37 °C in Luria Bertani (LB) broth with shaking at 220 rpm or on LB-agar plates (LB broth, 18 g l^–1^ agar). *E. coli* strain TOP10 was used for plasmid cloning. *E. coli* RL73 (Leenay et al, 2016) (BW25113 ΔCRISPR ΔLacOperon) was used for the VI-B CRISPR-Cas system expression. NEB® Turbo Competent *E. coli* cells were used for the MS2 phage plaquing assay. Antibiotics were added where appropriate at final concentrations of 100 μg/ml for ampicillin, 34 μg/ml for chloramphenicol, and 50 μg/ml for kanamycin for *E. coli*. *Porphyromonas gingivalis* ATCC 33277 was grown in ATCC 2722 medium at 37 °C in an anaerobic chamber.

### Nucleic-acid labeling

For the 5′ end labeling of RNA/DNA with ^32^P used for the Northern blotting/in-line probing, synthetic oligoribonucleotides/oligodeoxyribonucleotides (IDT) were treated with T4 PNK (New England Biolabs) in the presence of [gamma-^32^P]ATP (Hartmann Analytic). The labeled DNA was purified on a Sephadex G25 column, eluted in water. The labeled RNA was mixed with 2x RNA loading dye (0.025% bromophenol blue, 0.025% SDS, 0.025% xylene cyanol, 18 mM EDTA (pH 8.0), 93.64% formamide), separated on a 8% polyacrylamide gel with 8 M urea; the band with the RNA was excised from the gel, and the RNA was extracted from it using ZR small-RNA PAGE Recovery Kit (Zymo Research). For the 3′ end labeling of RNA with Cy5 used for EMSA, T7-transcripts were treated with T4 RNA ligase (Thermo Fisher Scientific) in the presence of pCp-Cy5 (Jena Bioscience). The labeled RNA was purified with RNA Clean and Concentrator columns-5 (Zymo Research).

### In-line probing

For the in-line probing sample, ~0.2 pmol ^32^P-5′ end-labeled RNA (ecrRNA, repeat, mecrRNA) was incubated for 40 h at room temperature in an in-line probing buffer (50 mM Tris-HCl, pH 8.3, 20 mM MgCl_2_, and 100 mM KCl). For alkaline ladders (OH), ~0.2 pmol ^32^P-5′ end-labeled RNA was denatured for 5 min at 95 °C in alkaline hydrolysis buffer (Ambion). For RNase T1 ladders, ~0.2 pmol ^32^P-5′ end-labeled RNA and 10 μg of yeast RNA (Ambion) was incubated in 1x sequencing buffer (Ambion) for 1 min at 95 °C, transferred on ice and further incubated with 0.1 U RNase T1 (Ambion) for 5 min at 37 °C. All reactions were prepared in 10 μl volume and were stopped by adding 10 μl colorless gel-loading solution (10 M urea, 1.5 mM EDTA, pH 8.0) on ice. The reactions were analyzed on 15% polyacrylamide gel with 8 M urea. The gels were dried, exposed to a storage phosphor screen, and analyzed using an Amersham Typhoon imaging system. The assay is performed in two replicates.

### RNA polymerase pausing assay

The DNA templates used for the in vitro transcription of the pre-crRNA with the native or mutated leader were amplified with the primers prAM464-5 from the plasmids pAM025 or pAM026 constructed by inserting the T7A1 promoter 35 bp upstream of the first repeat in the array (pAM250 or pAM254). To synchronize the transcription reactions, a halted complex was first formed. For that, the DNA templates (50 nM) were preincubated with 100 nM *E. coli* RNAP holoenzyme (New England Biolabs) in a 1x transcription buffer (20 mM MgCl_2_, 20 mM Tris-HCl (pH 8.0), 20 mM NaCl, 100 µM EDTA, and 14 mM ß-Mercaptoethanol) for 5 min at 37 °C, then the transcription was initiated with addition of 10 µM ApU dinucleotide, 25 µM rATP/rCTP, and 5% (v/v) [alpha-P^32^]rGTP (Hartmann Analytic) and further incubated for 10 min at 37 °C. The halted complex was purified to remove the radioactive nucleotides on a Sephadex G50 column, eluted in 1x transcription buffer. To transcribe the full-length pre-crRNA, 1 volume of the halted complex was mixed with 1 volume of the transcription mix (50 µM rNTPs, 0.6 mg/mL heparin, and 1x transcription buffer). Transcription aliquots were taken 10, 20, 30, 60, 90, 120, 180, and 300 s after the start of the full-size transcription and mixed with 2x RNA loading dye (0.025% bromophenol blue, 0.025% SDS, 0.025% xylene cyanol, 18 mM EDTA (pH 8.0), and 93.64% formamide) to stop the transcription. In addition, a chase sample was collected after 5 min of incubation, after the 300 s aliquot was taken with the addition of 500 µM rNTPs. The reactions were analyzed on 10% polyacrylamide gel with 7 M urea. The gels were dried, exposed to a storage phosphor screen, and analyzed using an Amersham Typhoon imaging system. The band sizes were determined with the help of RNase T1 digestion of the full-length transcript (chase sample). The assay was performed in two replicates.

### Total RNA extraction from bacteria

For total RNA extraction from *E. coli* or *Porphyromonas gingivalis* ATCC 33277, cells were grown to an OD_600_ of 0.8, 4 ml of culture was mixed with 1 ml of stop solution (95% EtOH + 5% phenol), and harvested with centrifugation at 5000 × *g* for 10 min at 4 °C. Cell pellet was resuspended in 600 µl 0.5 mg/ml lysozyme in TE pH 8.0; 60 µl 10% w/v SDS was added, the mix was incubated at 64 °C for 1 min. To the cell lysate 66 µl 3 M NaOAc, pH 5.2, and 750 µl phenol were added, the mix was incubated at 64 °C for 6 min. For the phase separation, the samples were centrifuged at 15,000 × *g* for 15 min at 4 °C. The aqueous layer was transferred into new tubes and mixed with 750 µl of chloroform. After another centrifugation at 15,000 × *g* for 15 min at 4 °C, the aqueous layer was mixed with 1.4 ml (30:1) EtOH: NaAcetate (from stock 3 M pH 6.5). After the samples were placed at −20 °C for RNA precipitation overnight, they were centrifuged at 15,000 × *g* for 30 min at 4 °C. The supernatant was removed, and the RNA pellet was washed with 70% EtOH and air-dried. RNA pellet was resuspended in water and cleaned with RNA Clean and Concentrator columns-25 (Zymo Research). For 5′ RACE, RNA was treated with TURBO DNase (Ambion).

### 5′ RACE

About 2 μg of DNase-treated total RNA extracted from *E. coli* containing pAM250 or *Porphyromonas gingivalis* ATCC 33277 was treated with 10U of T4 Polynucleotide kinase in 1x PNK buffer (New England Biolabs) with the addition of 10 nmol of ATP for 30 min at 37 °C to phosphorylate 5′-ends of the processed fraction of total RNA. The PNK-treated RNA was purified with phenol/chloroform/IAA, concentrated with RNA Clean and Concentrator columns-5 (Zymo Research) and treated with 7U of 5′ terminator exonuclease (TEX) (Epicentre) for 60 min at 30 °C to remove monophosphorylated transcripts. The TEX-treated RNA was purified with phenol/chloroform/IAA and concentrated with RNA Clean and Concentrator columns-5 (Zymo Research) and subjected to 5′ RACE using the Template Switching RT Enzyme Mix (New England Biolabs). Reverse transcription was performed with RT primer prAM146 (*E. coli*) or RT primer prAM157 (*P. gingivalis* ATCC 33277). To create a cDNA library, PCR was performed using the Q5 Hot Start High-Fidelity 2X Master Mix (New England Biolabs) with the PCR primers prAM139 and a TSO-specific primer (*E. coli*) or PCR primers prAM159 and a TSO-specific primer (*P. gingivalis* ATCC 33277). The cDNA libraries were purified using Zymo DNA Clean and Concentrator columns-5 (Zymo Research), cloned into the supplied linearized vector pMiniT 2.0 using NEB PCR Cloning Kit (New England Biolabs). 16 random colonies were picked for colony PCR with the primers prCL1504 and prCL1505 to determine the sequence of the plasmid insert, purified using Zymo DNA Clean and Concentrator columns-5 (Zymo Research) and submitted for Sanger sequencing.

### PgiCas13b protein purification

The vector pAM350 was transformed into *E. coli* BL21 codon+ RIL. The culture was grown in LB medium with kanamycin and chloramphenicol to OD_600_ = 0.6, then the expression of *cas13b* was induced with 0.25 mM IPTG and the induced culture was growing at 18 °C overnight. After the cells were harvested, the pellet was resuspended in a lysis buffer (50 mM HEPES, pH 7.5, 500 mM NaCl, and 0.5 mM DTT) containing DNase and protease inhibitors and lysed by sonication. The lysate was clarified by centrifugation for 30 min at 30,000 × *g*, 4 °C. Clarified lysate was added to the HisTrap crude FF 5 ml column equilibrated to the lysis buffer. The column was washed with lysis buffer, then with the IMAC wash buffer I (50 mM HEPES, pH 7.5, 1000 mM NaCl, 0.5 mM DTT), IMAC wash buffer II (20 mM HEPES, pH 7.5, 400 mM NaCl, and 2 mM DTT). SenP2 Sumo protease resuspended in IMAC wash buffer II was loaded onto the column, the column was sealed and incubated overnight at 6–8 °C. The column was washed in IMAC wash buffer II, and the protein was eluted in IMAC elution buffer (20 mM HEPES, pH 7.5, 400 mM NaCl, 0.5 mM DTT, and 250 mM imidazole). Eluate was applied to a HiTrap Heparin 5 ml ion exchange chromatography column, washed with IEX buffer A (20 mM HEPES, pH 7.0, 400 mM NaCl, and 2 mM DTT) and eluted with a linear gradient to IEX buffer B (20 mM HEPES, pH 7.0, 2000 mM NaCl, and 2 mM DTT). The eluate fractions containing the protein were loaded on a size exclusion chromatography (SEC) HiLoad Superdex 200 16/600 pg column, equilibrated in SEC buffer (10 mM HEPES, pH 7.0, 500 mM NaCl, and 2 mM DTT). The fractions containing PgiCas13b were concentrated and stored at −80 °C.

### In vitro T7 transcription

For the in vitro processing assay and EMSA, RNA was synthesized using HiScribe T7 High Yield RNA Synthesis Kit (New England Biolabs). The T7 transcription templates were produced by PCR with the primers prAM185 and prAM191 using pAM250, pAM254, and pAM272 as the templates for the PCRs. The transcription was purified with RNA Clean and Concentrator columns-5 (Zymo Research), treated with TURBO DNase (Ambion) and cleaned again with RNA Clean and Concentrator columns-5 (Zymo Research).

### EMSA

For the electrophoretic mobility shift assay (EMSA), 20 nM Cy5-labeled RNA was incubated with various amounts of purified PgiCas13b from 11 nM to 3 μM for 15 min at room temperature in a binding buffer (10 mM Tris-HCl, pH 7.5, 50 mM NaCl, 0.1% BSA, 10 mM EDTA, and 5% glycerol) in the presence of 10 μg of yeast RNA (#AM2283, Ambion) to enhance specific RNA–protein interaction. The samples were separated on 2% agarose-TBE gels at 4 °C. Gels were visualized using an Amersham Typhoon imaging system. RNA–protein gel shifts were quantified using ImageQuant TL and plotted in GraphPad Prism version 10. The bands running at the size of the RNA alone were used to calculate the percentage of the unbound RNA. Regression analysis and Kd calculation were performed in Prism 10 using nonlinear fit with one-site specific binding. The assay is performed in three replicates.

### In vitro processing assay

In vitro processing assay was performed with 200 ng of T7-transcripts and 16 pmol of purified PgiCas13b for 15 min at 37 °C in a nuclease buffer (10 mM Tris HCl, pH 7.5, 50 mM NaCl, 0.5 mM MgCl_2_, and 0.1% BSA). The reactions were stopped by the addition of 2x RNA loading dye (0.025% bromophenol blue, 0.025% SDS, 0.025% xylene cyanol, 18 mM EDTA (pH 8.0), and 93.64% formamide) and analyzed on 6% polyacrylamide gel with 8 M urea. The gel was stained with SYBR Green II (Biozym) and visualized using an Amersham Typhoon imaging system.

### Plasmid interference assay

About 50 ng of plasmid encoding the transcribed target flanked with protospacer-flanking sequences (PFS) (AmpR) was electroporated into *E. coli* cells harboring the plasmid encoding the type VI-B system (KanR). After recovering for 1 h in SOC medium (20 g/L tryptone, 5 g/L yeast extract, 3.6 g/L glucose, 0.5 g/L NaCl, 0.186 g/L KCl, 0.952 g/L MgCl2, PH 7.0) at 37 °C with shaking at 220 rpm, cells were serially diluted in 1x PBS, and 5-μL droplets were plated on LB-agar plates with ampicillin and kanamycin. Colony numbers were recorded for analysis after 16 h of growth. The assay is performed in three biological replicates.

### Northern blotting

About 10 μg of total RNA extracted from *E. coli* was mixed with 2x RNA loading dye (0.025% bromophenol blue, 0.025% SDS, 0.025% xylene cyanol, 18 mM EDTA (pH 8.0), and 93.64% formamide), denatured at 95 °C for 5 min, quickly placed on ice and loaded on 8% polyacrylamide gel with 8 M urea. RNA was transferred from a gel onto Hybond-XL membranes (Amersham Hybond-XL, GE Healthcare) using an Electroblotter with an applied voltage of 50 V for 1 h at 4 °C (Tank-Elektroblotter Web M, PerfectBlue), crosslinked twice with 120 mJ/cm^2^ UV-light (UV-lamp T8C; 254 nm, 8 W), hybridized overnight in Roti-Hybri-Quick buffer at 42 °C with ^32^P-5′ end-labeled oligodeoxyribonucleotides. After washing first with 5x and then with 1x SSC buffer, the membrane was exposed to a storage phosphor screen and visualized using an Amersham Typhoon imaging system. The assay was performed in three biological replicates. crRNAs band intensities were quantified using ImageQuant TL and plotted in GraphPad Prism version 10.

### Plaque formation assay in *E. coli*

In the plasmid pAM277 encoding the native Type VI-B system, the spacer 1 was replaced with the spacer targeting the MS2 phage. Two different plasmids were transformed into NEB® Turbo competent *E. coli* cells: the plasmid pAM277 (KanR) and a plasmid expressing in *trans* either ecrRNA (pAM417) or mecrRNA (pAM418) (AmpR). After the transformation, the cells were plated onto LB-agar plates containing the appropriate antibiotics. Following incubation at 37 °C overnight, three single colonies (three biological replicates) were inoculated overnight at 37 °C in LB-antibiotic medium. The overnight cultures were diluted to OD_600_ = 0.05 and grown to OD_600_ ≈ 0.5 at 37 °C in 100 ml LB-antibiotic medium. The cells were harvested once by centrifugation at 4000 rpm for 10 min at room temperature, the supernatant was removed, and the cell pellet was resuspended in 10 ml of LB-antibiotic medium. About 750 μl of the cells were mixed with 4 ml of the “soft” agar (10 g/L tryptone, 5 g/L yeast extract, 5 g/L NaCl, 7.5 g/L agar) containing the appropriate antibiotics preheated to 60 °C in advance and 10 μl of the MS2 phage dilutions. The cell-agar mix was poured onto LB-antibiotic agar plates (24-ml LB-antibiotic agar in Petri dishes with the following dimensions: d = 90 mm, h = 16.2 mm). Tenfold serial dilutions of the MS2 phage were prepared in LB medium. The lowest dilution of the MS2 phage was 4.8 × 10^8^ PFU/mL, the highest dilution was 4.8 × 10^5^ PFU/mL. After the plates were incubated at 37 °C overnight, the following morning, the plaques were counted on the plates with the dilutions 4.8 × 10^7^ (for ecrRNA) or 4.8 × 10^5^ PFU/mL (for mecrRNA).

To assess whether the MS2-targeting spacer protects cells from infection, we compared plaque formation in *E. coli* strains carrying the native array or an array with spacer 1 targeting MS2. A plasmid with the native array (pAM250) (KanR) or a plasmid with spacer 1 targeting MS2 (pAM277) (KanR) were transformed into NEB® Turbo competent *E. coli* cells. The overnight cultures were diluted to OD_600_ = 0.05 and grown to OD_600_ ≈ 0.5 at 37 °C in 10 ml LB-Kan medium. The cells were harvested once by centrifugation at 4000 rpm for 10 min at room temperature, the supernatant was removed, and the cell pellet was resuspended in 1 ml of LB-Kan medium. About 750 μl of the cells were mixed with 4 ml of the “soft” agar containing Kan. The cell-agar mix was poured onto LB-Kan agar plates. Tenfold serial dilutions of the MS2 phage were prepared in LB medium. The lowest dilution of the MS2 phage was 1.3 × 10^9^ PFU/mL, the highest dilution was 1.3 × 10^5^ PFU/mL. About 3 µL of the phage dilutions were spotted onto the agar using a multichannel pipette. After the plates were incubated at 37 °C overnight, the following morning, the photos of the plates were taken. The assay was performed in three biological replicates.

## Supplementary information

Appendix

Table EV1

Table EV2

Table EV3

Table EV4

Peer Review File

Dataset EV1

Dataset EV2

Source data Fig. 3

Source data Fig. 4

Source data Fig. 5

Source data Fig. 6

Expanded View Figures

## Data Availability

Source data are provided. This study includes no data deposited in external repositories. The source data of this paper are collected in the following database record: biostudies:S-SCDT-10_1038-S44318-026-00769-1.
