# Supplementary material for: A leader-repeat hairpin blocks extraneous CRISPR RNA production in diverse CRISPR-Cas13 systems
Source: EMBO J. 2026 Apr 2;45(10):3396–415. doi: 10.1038/s44318-026-00769-1 (PMC13187072; doi:10.1038/s44318-026-00769-1)
Supplement: Supplementary file 1 — Appendix [file 44318_2026_769_MOESM1_ESM.pdf]

Appendix for

A leader-repeat hairpin blocks extraneous CRISPR RNA production in diverse CRISPR-Cas13 systems

Appendix Figure S1 2
Appendix Figure S2 4
Appendix Figure S3 5

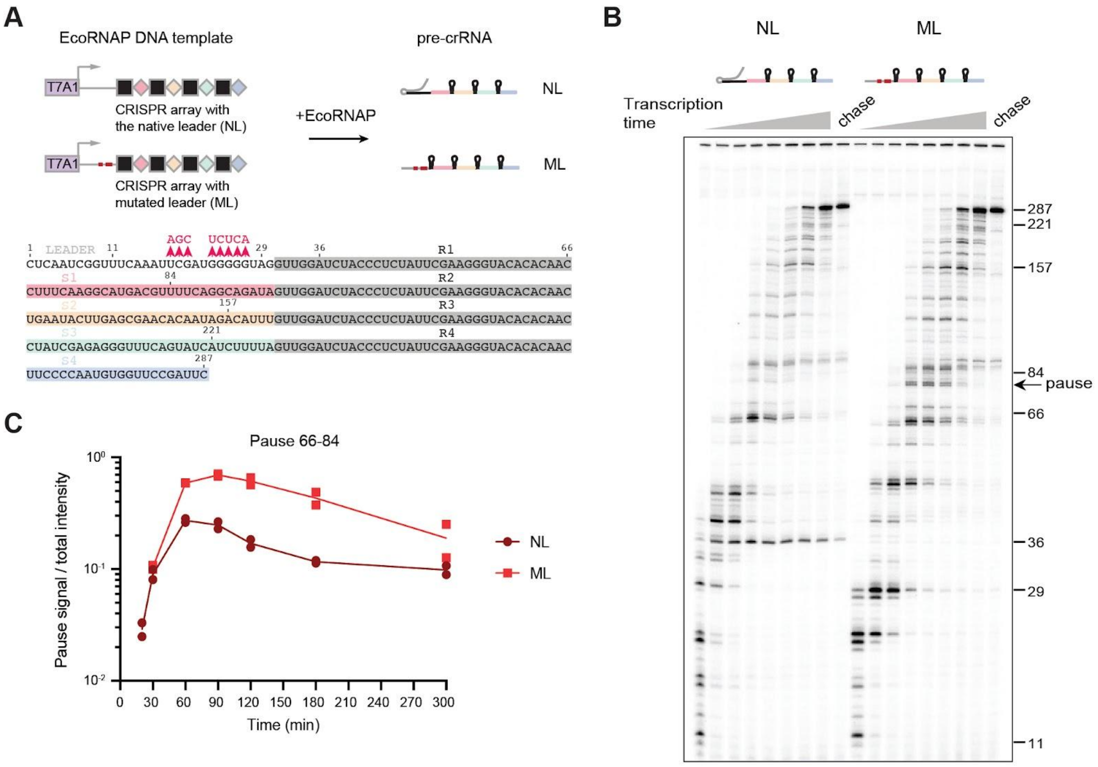

Appendix Figure S1. Mutating the native leader increases RNAP pausing upon transcription of the first repeat in *P. gingivalis* ATCC 33277.

A. dsDNA templates used for the *in vitro* transcription by EcoRNAP of the pre-crRNAs with the native leader or mutated leader. Sequence of the pre-crRNA transcript synthesized by the EcoRNAP. Mutations in the leader designed to restore the hairpin in the first repeat (R1) are in red.

B. Transcription of the pre-crRNA with the native leader (NL) or mutated leader (ML) by EcoRNAP. Transcription was stopped by taking the aliquots of the reaction mix at varying time points after its start and mixing them with the denaturing gel loading buffer. ^{32}P-labeled RNA was separated on a 10% polyacrylamide gel with 7M urea. The numbers on the right side of the gel correspond to the last nucleotide of each transcript, the numbering of which in the pre-crRNA sequence is depicted in A. RNAP pausing between the 66 and 84 nt of the transcript occurs downstream of R1 transcription. The loss of pausing at nt position 36 in ML occurs in the region adjacent to the mutations. The displayed gel is representative of duplicate independent experiments.

C. Calculation of the band intensities in the pause site 66-84 normalized to the total intensity of the lane. Two independent measurements and their mean were plotted.

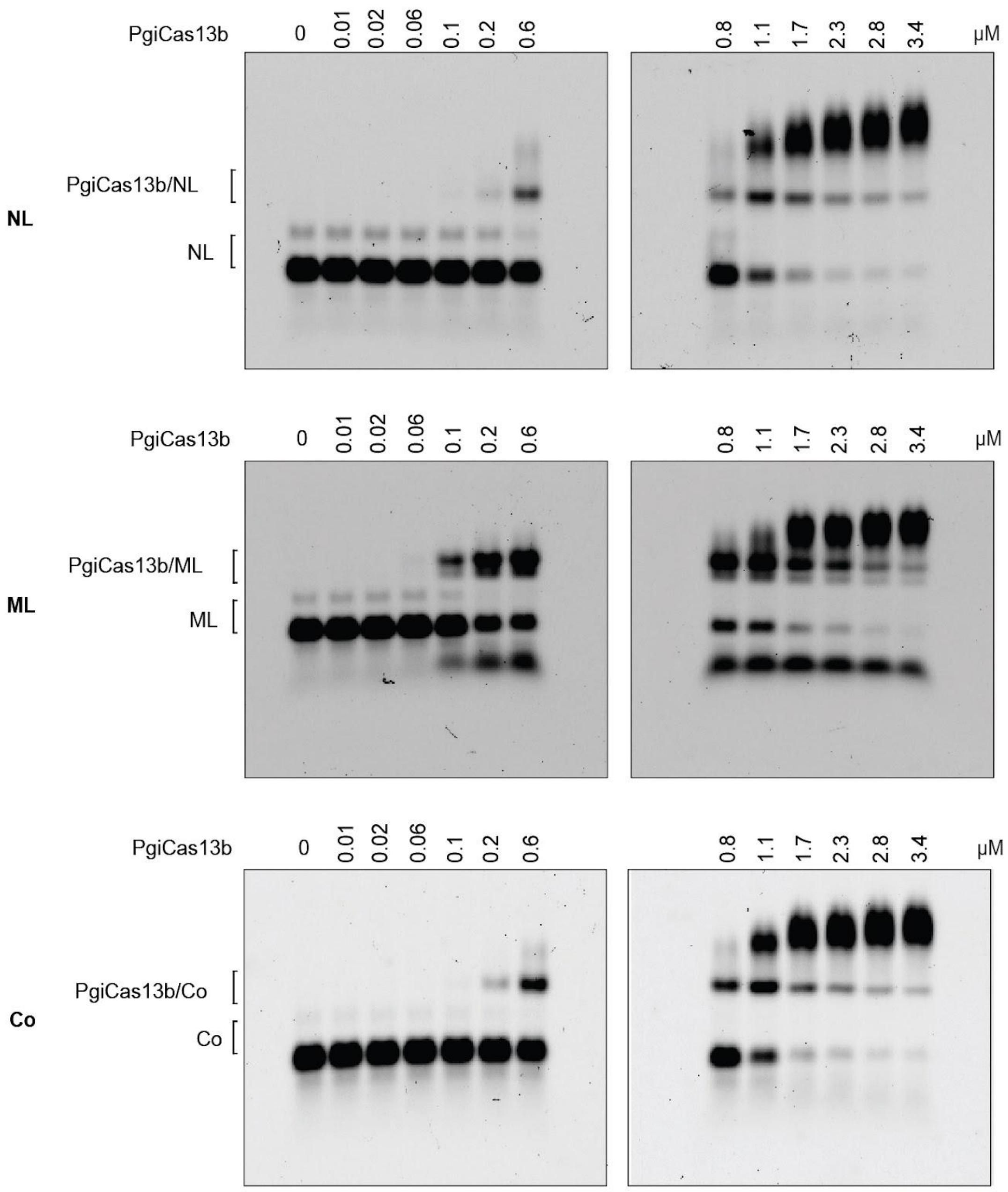

Appendix Figure S2. Binding of the ecrRNA by Cas13b is inhibited by the leader-repeat hairpin *in vitro*.

Electrophoretic mobility shift assay (EMSA) gels showing the affinity of PgiCas13b to the 3′-labeled pre-crRNAs (NL, ML, Co). The experiment was performed with three independent replicates. One representative 2% agarose gel is shown. The bands running at the size of the RNA alone were used to calculate the percentage of the unbound RNA.

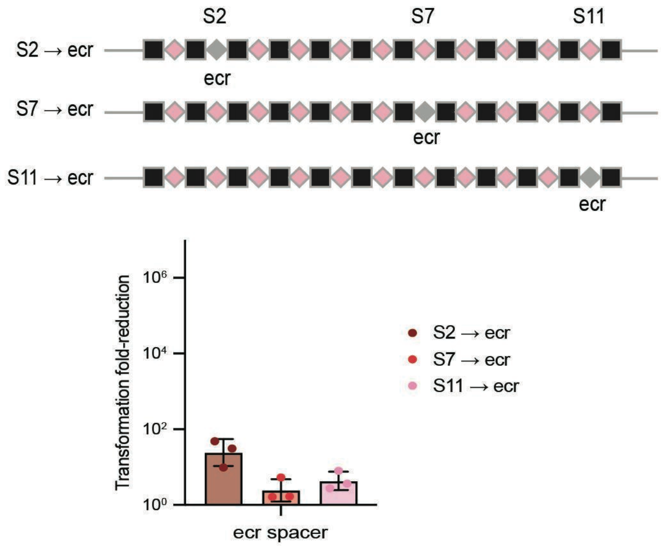

Appendix Figure S3. The ecrRNA-matching spacer leads to plasmid clearance when replacing spacers on the native array.

Plasmid interference assay. The targets of ecrRNA (ecr) or no-target control were co-transformed with a plasmid encoding the VI-B system from *P. gingivalis* AJW4 containing the array, in which the spacer S2, S7, or S11 was swapped with the 30 nts of the leader RNA fragment proximal to the array (ecr). The transformation was plated on double antibiotic selection plates. The experiment was performed in three biological replicates. On the plot, each dot represents an independent biological replicate, while the bars and error bars represent the geometric mean and geometric standard deviation.
