# Supplementary figures and images for: A leader-repeat hairpin blocks extraneous CRISPR RNA production in diverse CRISPR-Cas13 systems
Source: EMBO J. 2026 Apr 2;45(10):3396–415. doi: 10.1038/s44318-026-00769-1 (PMC13187072; doi:10.1038/s44318-026-00769-1)

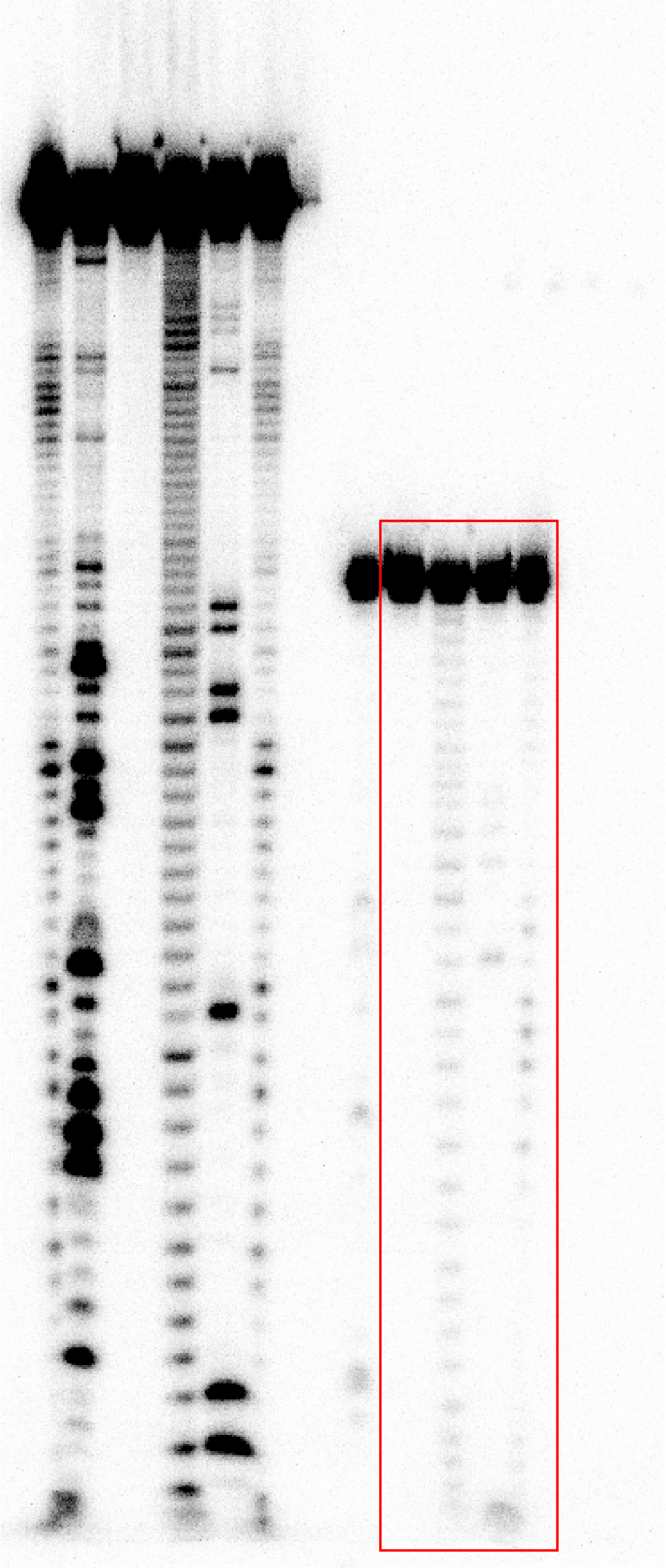

Supplement: Supplementary file 9 — Source data Fig. 3 [file 44318_2026_769_MOESM9_ESM.zip › EMBOJ-2025-121651_Source_files_Figure 3/Figure 3C.png]

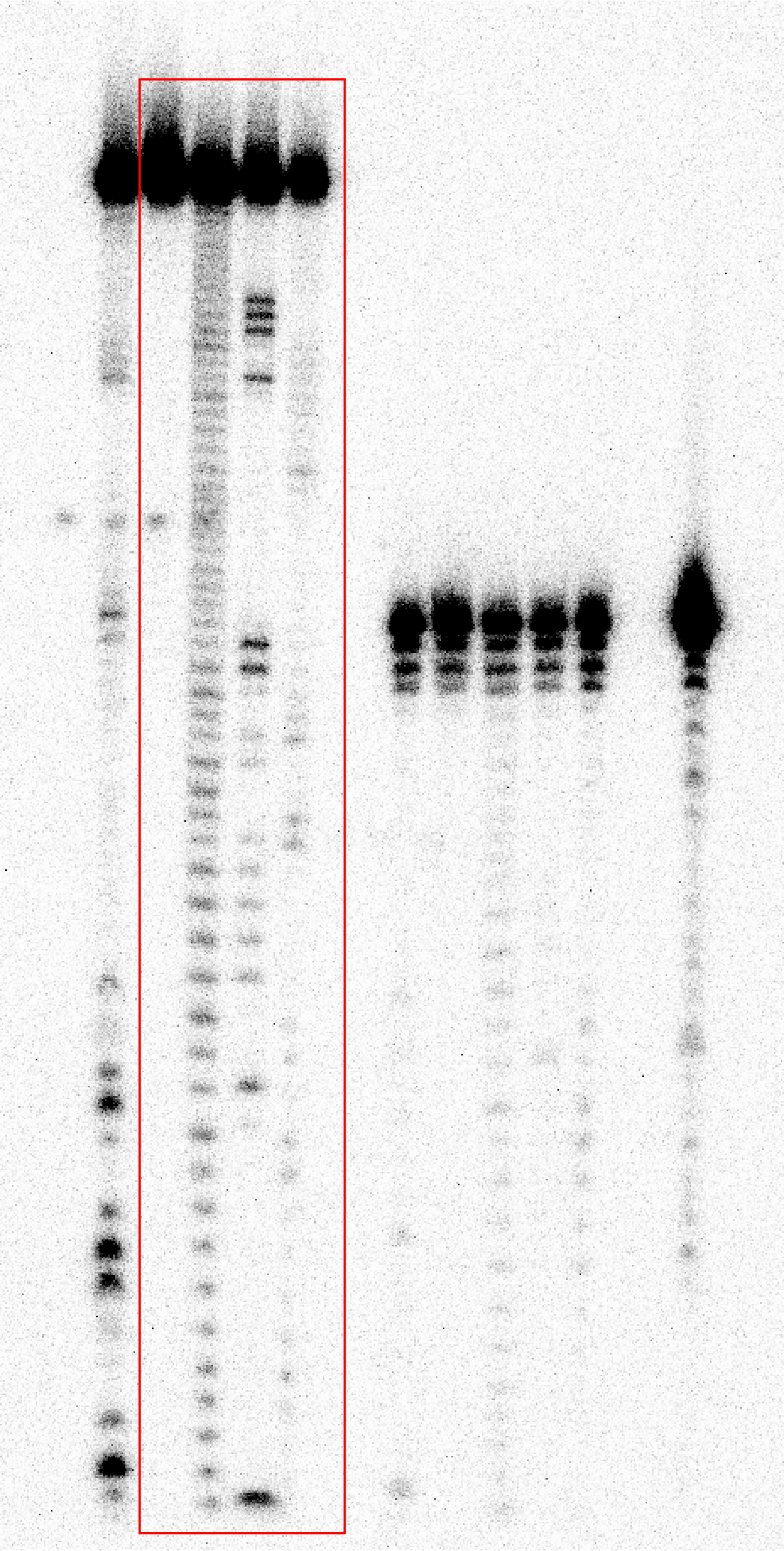

Supplement: Supplementary file 9 — Source data Fig. 3 [file 44318_2026_769_MOESM9_ESM.zip › EMBOJ-2025-121651_Source_files_Figure 3/Figure 3B.png]

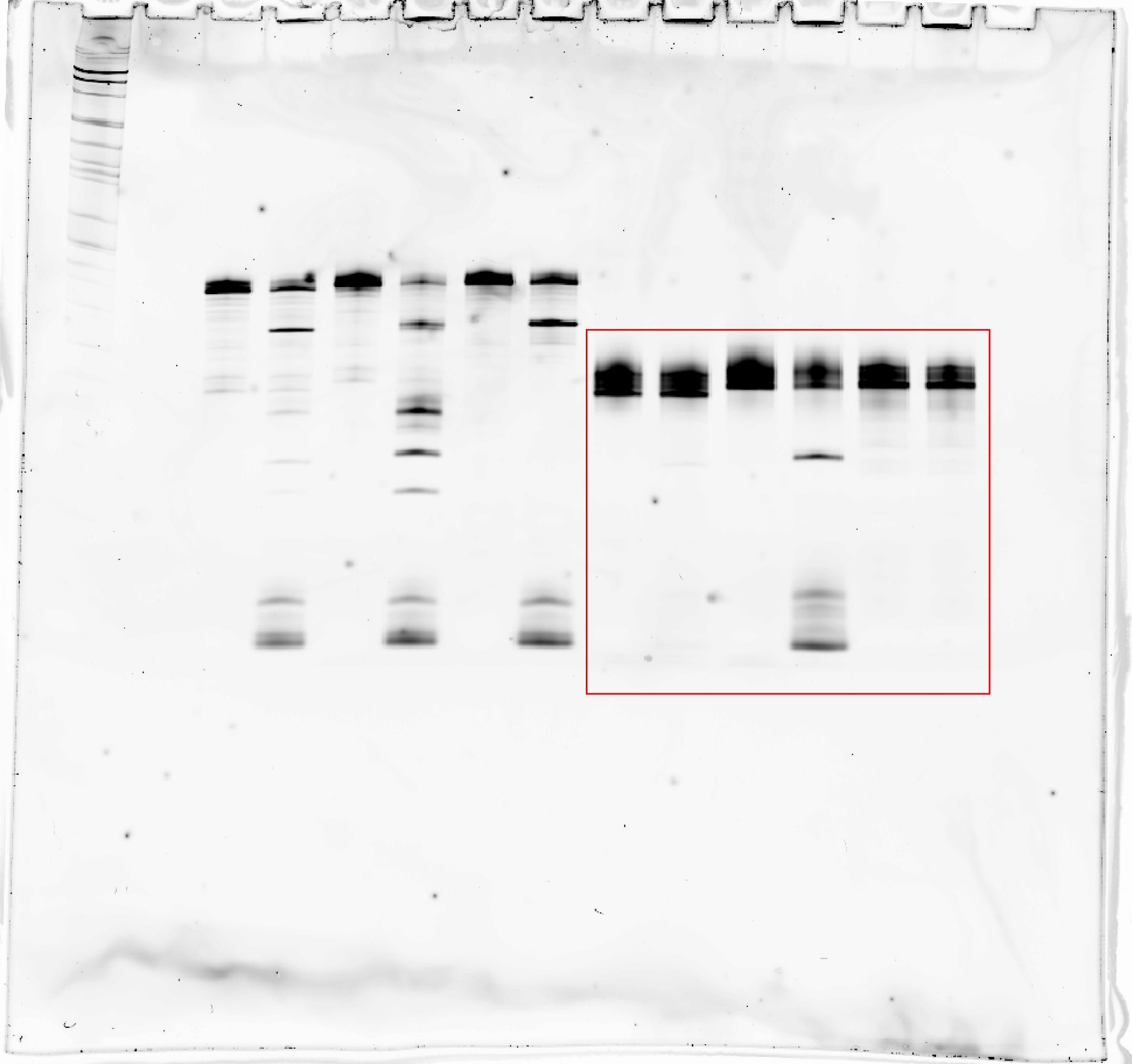

Supplement: Supplementary file 10 — Source data Fig. 4 [file 44318_2026_769_MOESM10_ESM.zip › EMBOJ-2025-121651_Source_files_Figure 4/Figure 4C.png]

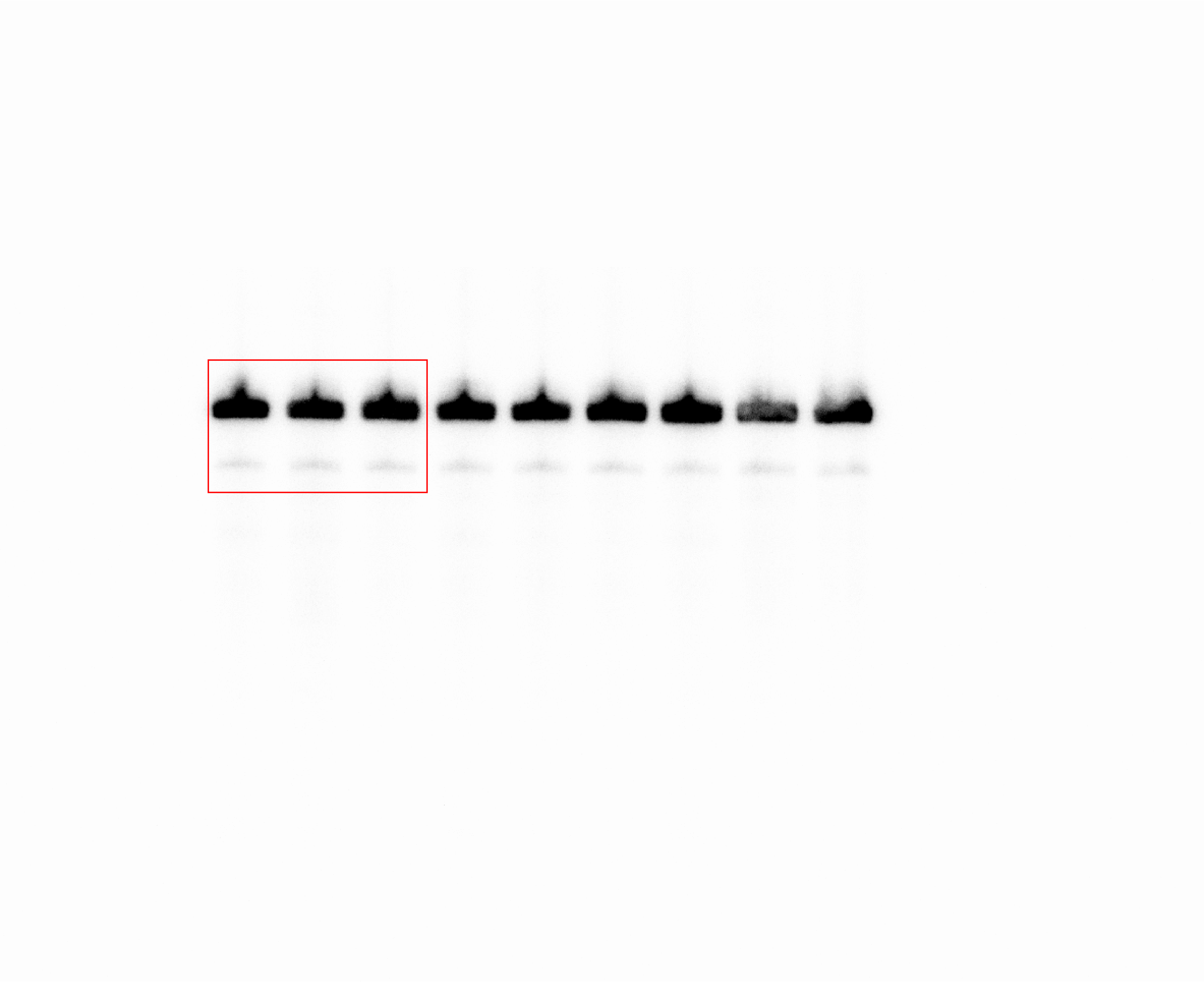

Supplement: Supplementary file 11 — Source data Fig. 5 [file 44318_2026_769_MOESM11_ESM.zip › EMBOJ-2025-121651_Source_files_Figure 5/Figure 5B_5S.png]

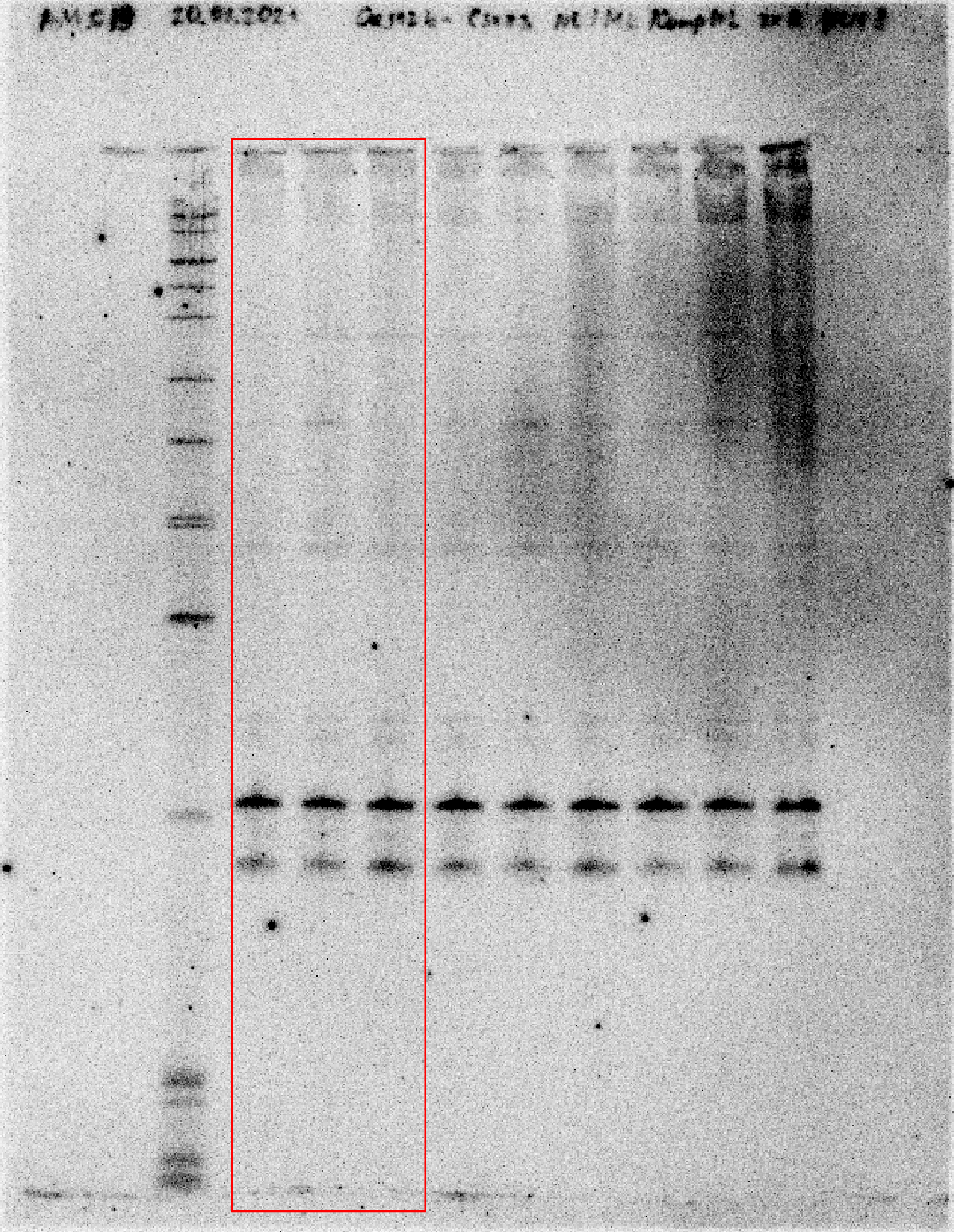

Supplement: Supplementary file 11 — Source data Fig. 5 [file 44318_2026_769_MOESM11_ESM.zip › EMBOJ-2025-121651_Source_files_Figure 5/Figure 5B_S11.png]

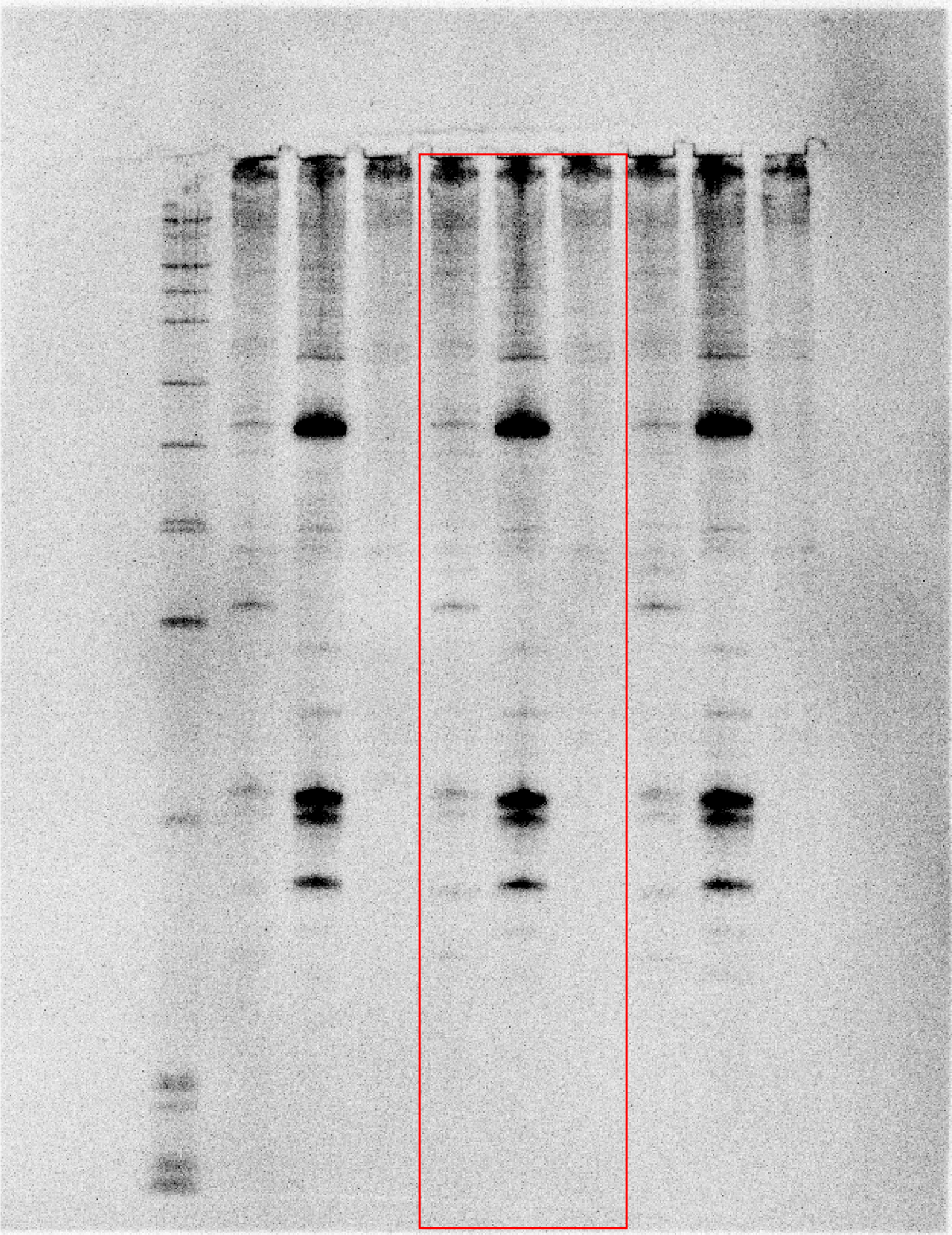

Supplement: Supplementary file 11 — Source data Fig. 5 [file 44318_2026_769_MOESM11_ESM.zip › EMBOJ-2025-121651_Source_files_Figure 5/Figure 5B_ecr-mecr.png]

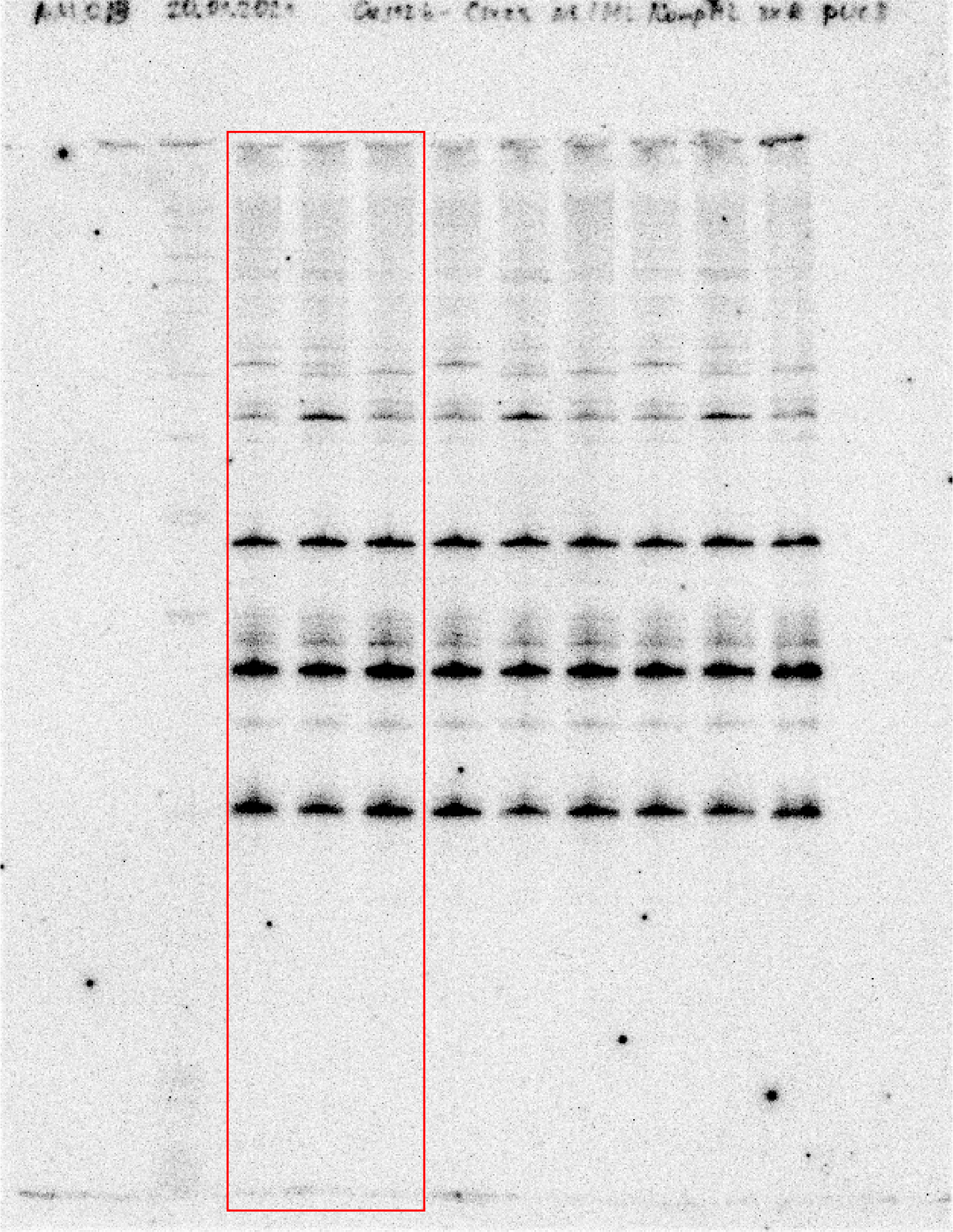

Supplement: Supplementary file 11 — Source data Fig. 5 [file 44318_2026_769_MOESM11_ESM.zip › EMBOJ-2025-121651_Source_files_Figure 5/Figure 5B_S3.png]

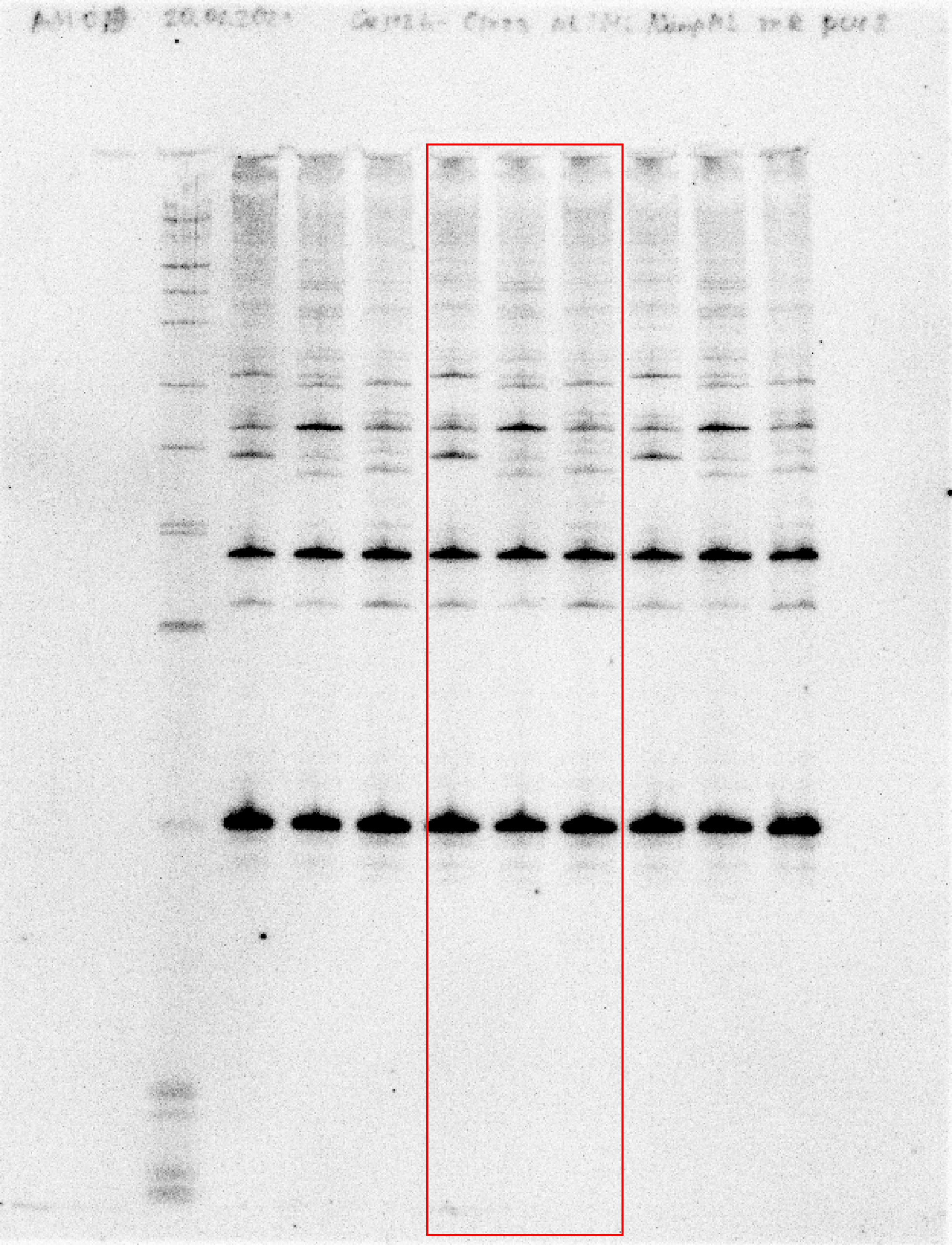

Supplement: Supplementary file 11 — Source data Fig. 5 [file 44318_2026_769_MOESM11_ESM.zip › EMBOJ-2025-121651_Source_files_Figure 5/Figure 5B_S2.png]

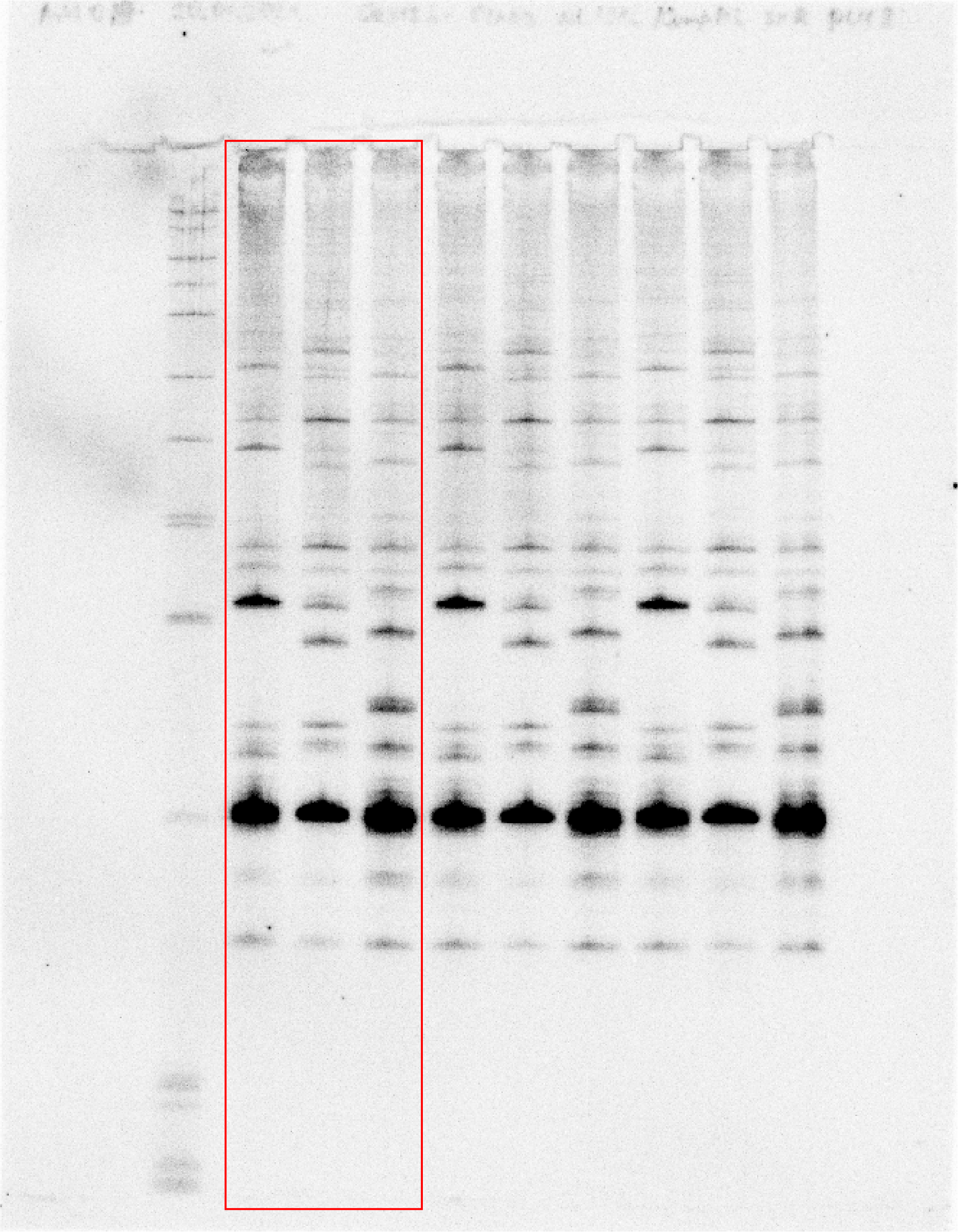

Supplement: Supplementary file 11 — Source data Fig. 5 [file 44318_2026_769_MOESM11_ESM.zip › EMBOJ-2025-121651_Source_files_Figure 5/Figure 5B_S1.png]

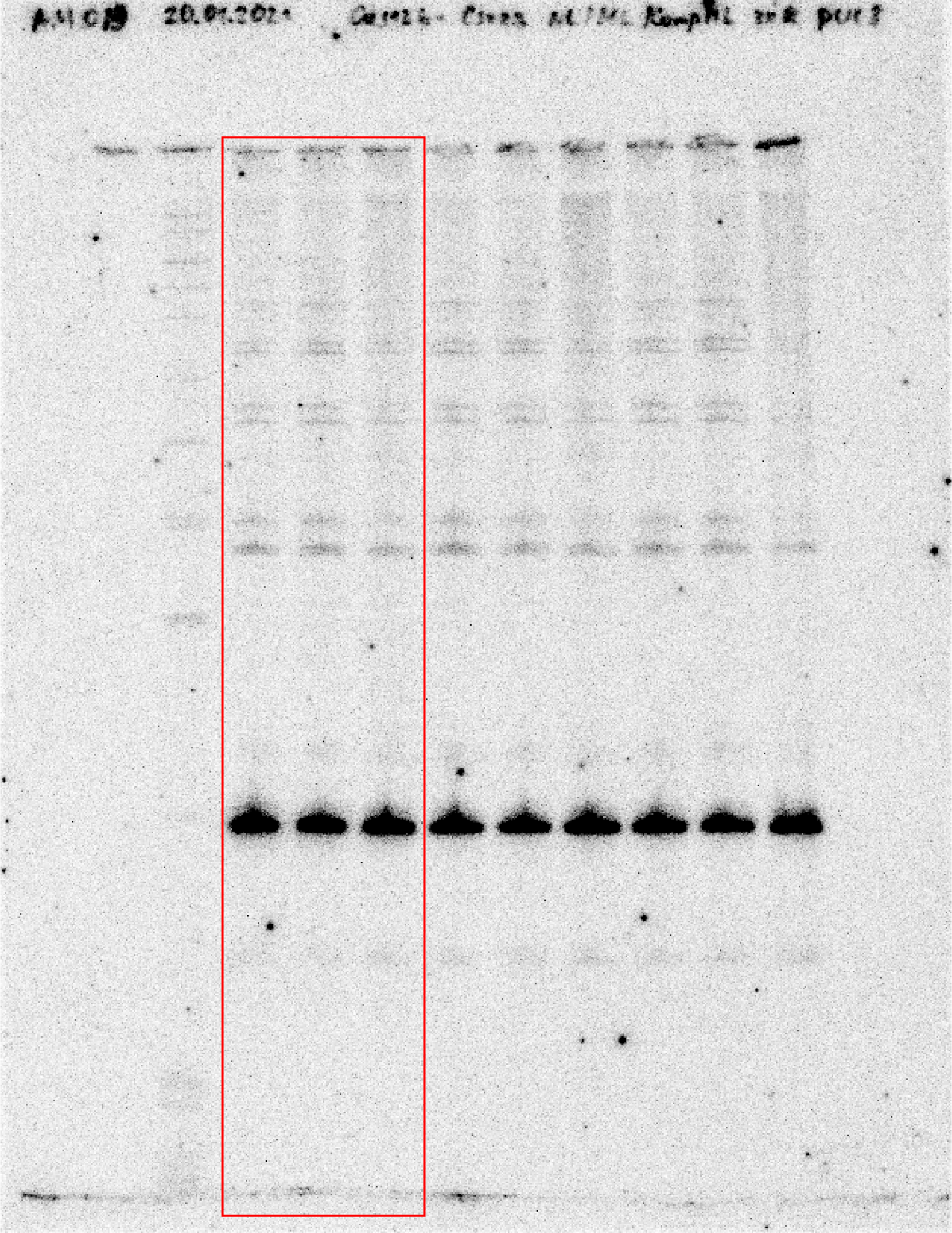

Supplement: Supplementary file 11 — Source data Fig. 5 [file 44318_2026_769_MOESM11_ESM.zip › EMBOJ-2025-121651_Source_files_Figure 5/Figure 5B_S4.png]

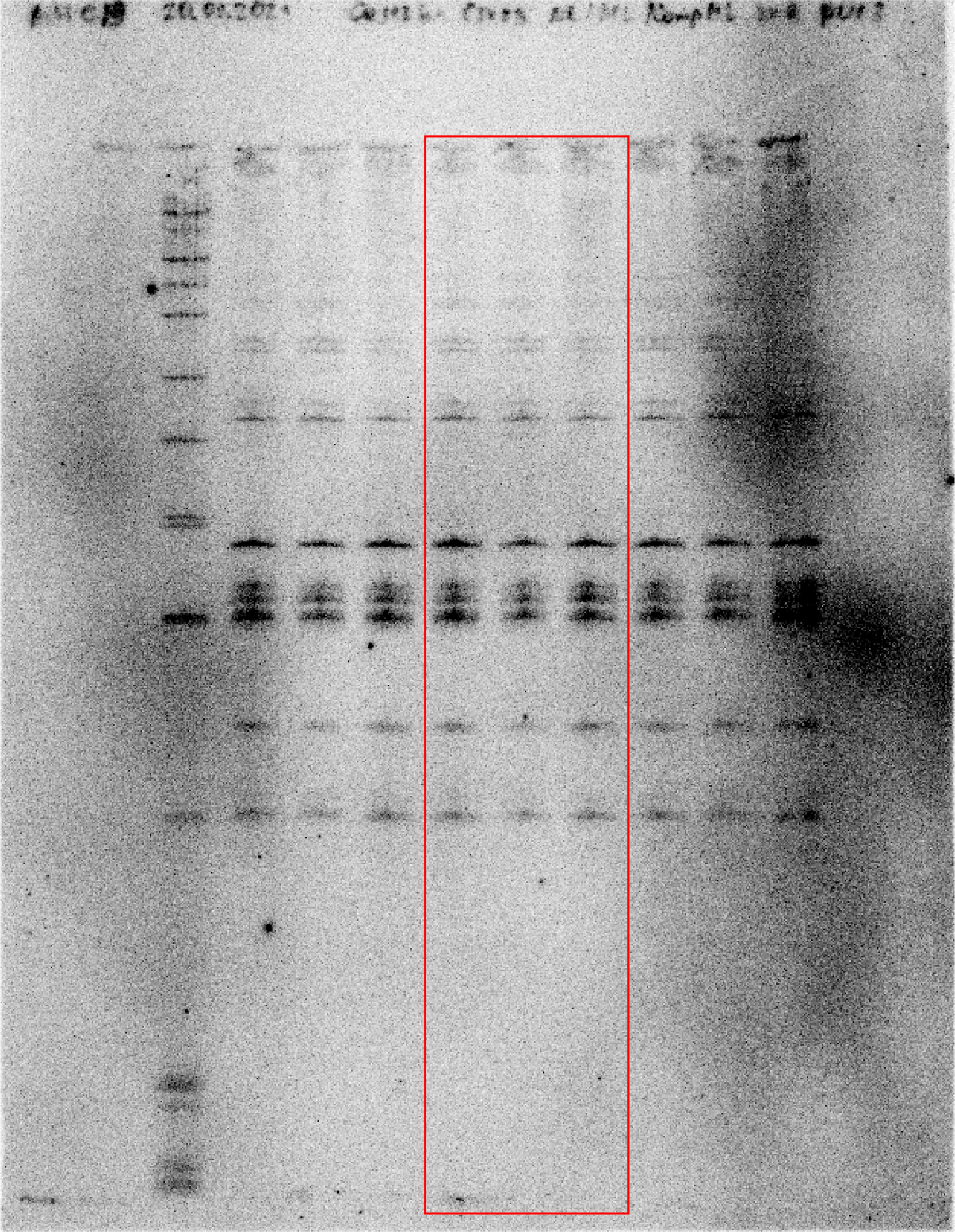

Supplement: Supplementary file 11 — Source data Fig. 5 [file 44318_2026_769_MOESM11_ESM.zip › EMBOJ-2025-121651_Source_files_Figure 5/Figure 5B_S7.png]
