# Supplementary material for: A leader-repeat hairpin blocks extraneous CRISPR RNA production in diverse CRISPR-Cas13 systems
Source: EMBO J. 2026 Apr 2;45(10):3396–415. doi: 10.1038/s44318-026-00769-1 (PMC13187072; doi:10.1038/s44318-026-00769-1)
Supplement: Supplementary file 13 — Expanded View Figures [file 44318_2026_769_MOESM13_ESM.pdf]

Expanded View Figures

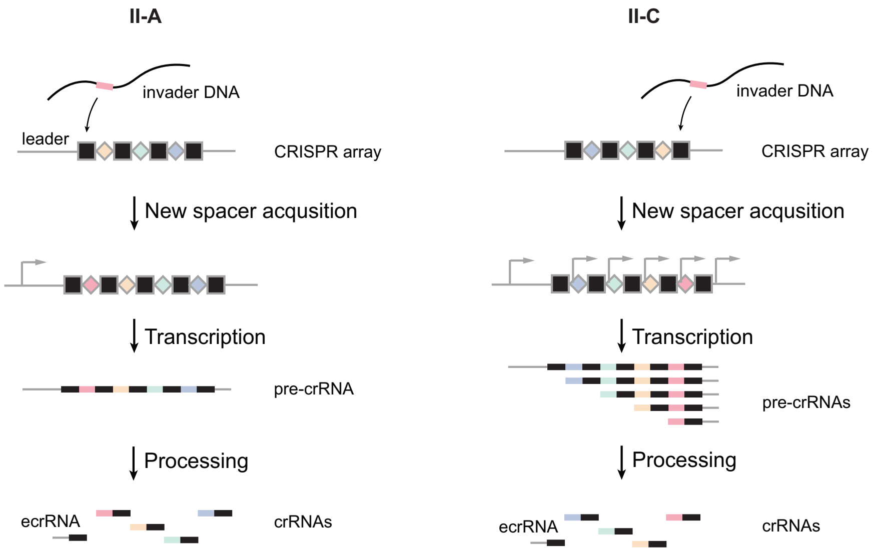

Figure EV1. II-A and II-C CRISPR-Cas systems would both derive an extraneous crRNA from the first repeat adjacent to the newest spacer (II-A) or to the oldest spacer (II-C).

The newest spacer is acquired through the first repeat in II-A systems or the last repeat in II-C systems. The CRISPR array is transcribed as a single pre-crRNA (II-A) or as multiple pre-crRNAs due to the repeats containing promoters (II-C). The ecrRNA would be formed at the beginning of the array from the first repeat adjacent to the newest spacer (II-A) or to the oldest spacer (II-C).

 © The Author(s)

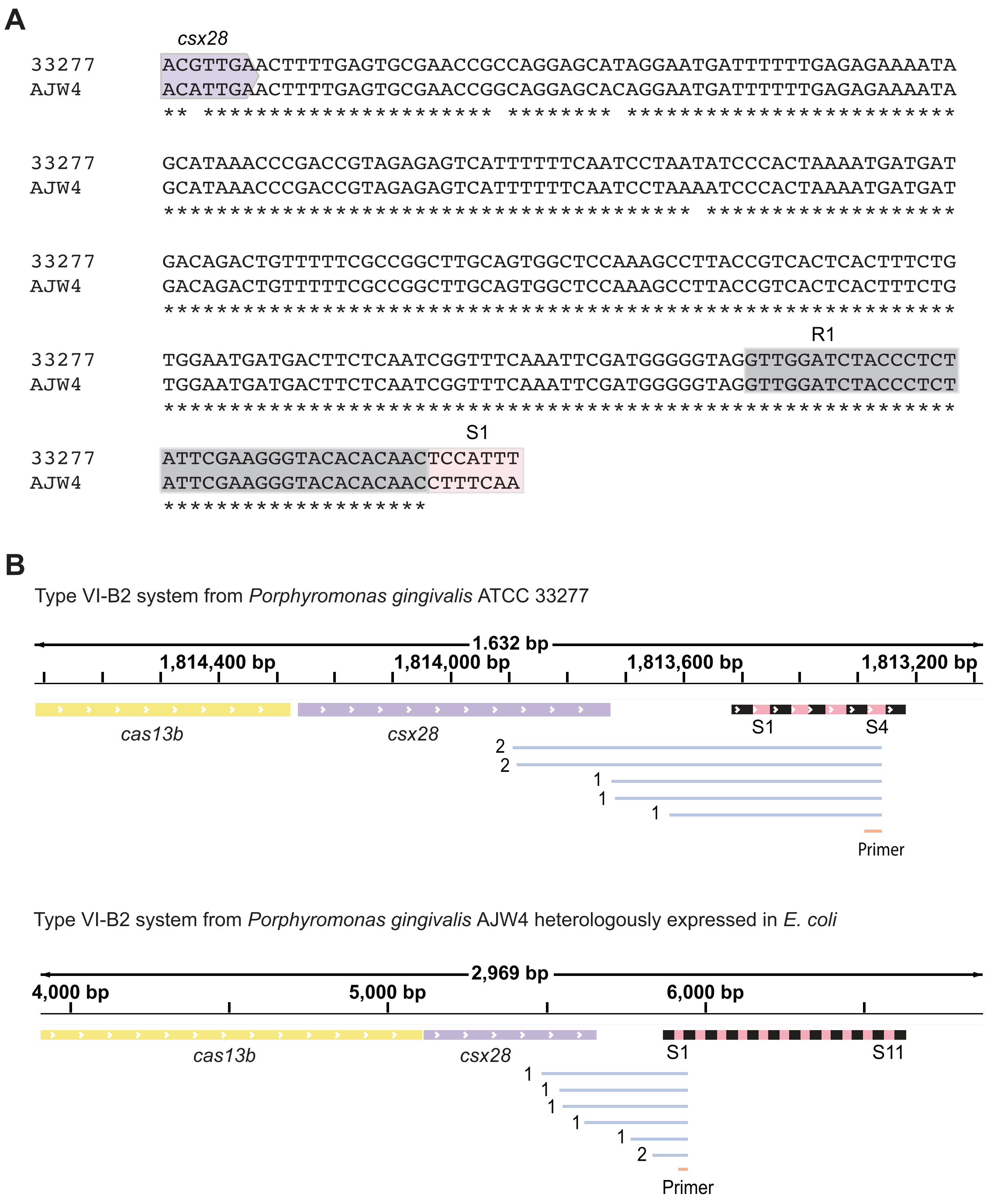

Figure EV2. Transcription of the CRISPR array in the *P. gingivalis* VI-B2 system is initiated far upstream of the array.

(**A**) Alignment of the first repeat and the sequence upstream of it in *P. gingivalis* ATCC 33277 and *P. gingivalis* AJW4. The first repeat and the upstream portion forming the leader-repeat stem-loop is identical between the two. (**B**) Alignment of the primary transcripts of the array (in blue) obtained through 5′ RACE to the Type VI-B system. The number of the sequenced primary transcripts with the same 5′ sequence is shown at the beginning of the transcript. The RNA subjected to 5′ RACE was extracted from *P. gingivalis* ATCC 33277 (NC_010729.1) (top) or from the *E. coli* strain heterologously expressing the VI-B system from *P. gingivalis* AJW4 on the plasmid pAM250 (bottom). The primer used for the PCR amplification of the transcripts converted into cDNA is in orange.

© The Author(s)

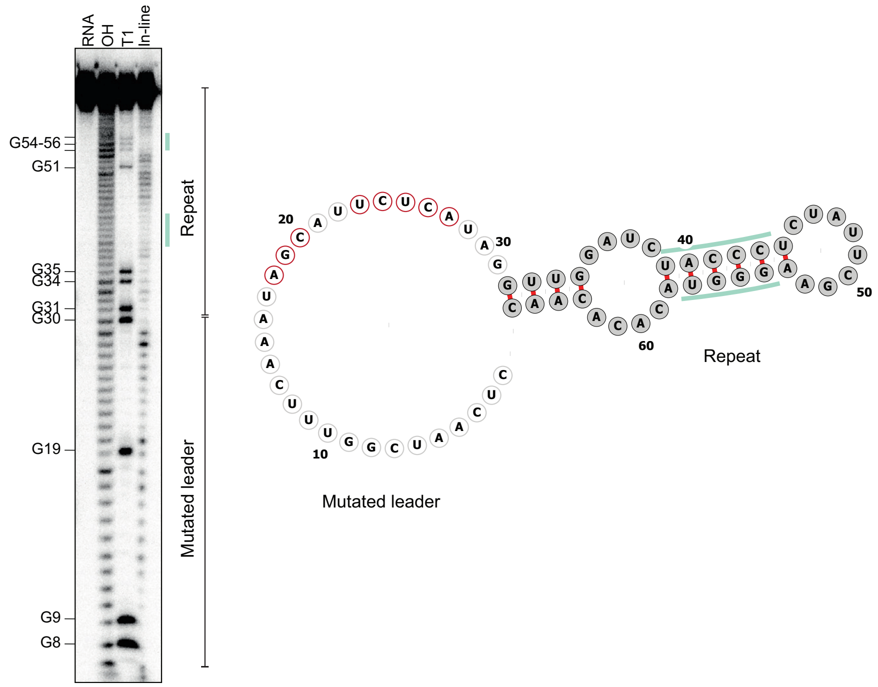

Figure EV3. Mutating the leader disrupting the leader-repeat stem-loop restores the internal hairpin in the repeat.

In-line probing of ~0.2 pmol P^{32}-labeled mutated ecrRNA (mecrRNA). Spontaneous cleavage of single-stranded regions was analyzed on a 10% polyacrylamide gel with 7 M urea. Untreated RNA (lane RNA), partially alkali (lane OH) or RNase T1 (lane T1) digested RNAs served as ladders. The double-stranded nucleotides are marked with green vertical bars. The displayed gel is representative of duplicate independent experiments. The MFE structure is computationally predicted with RNAfold.

 © The Author(s)

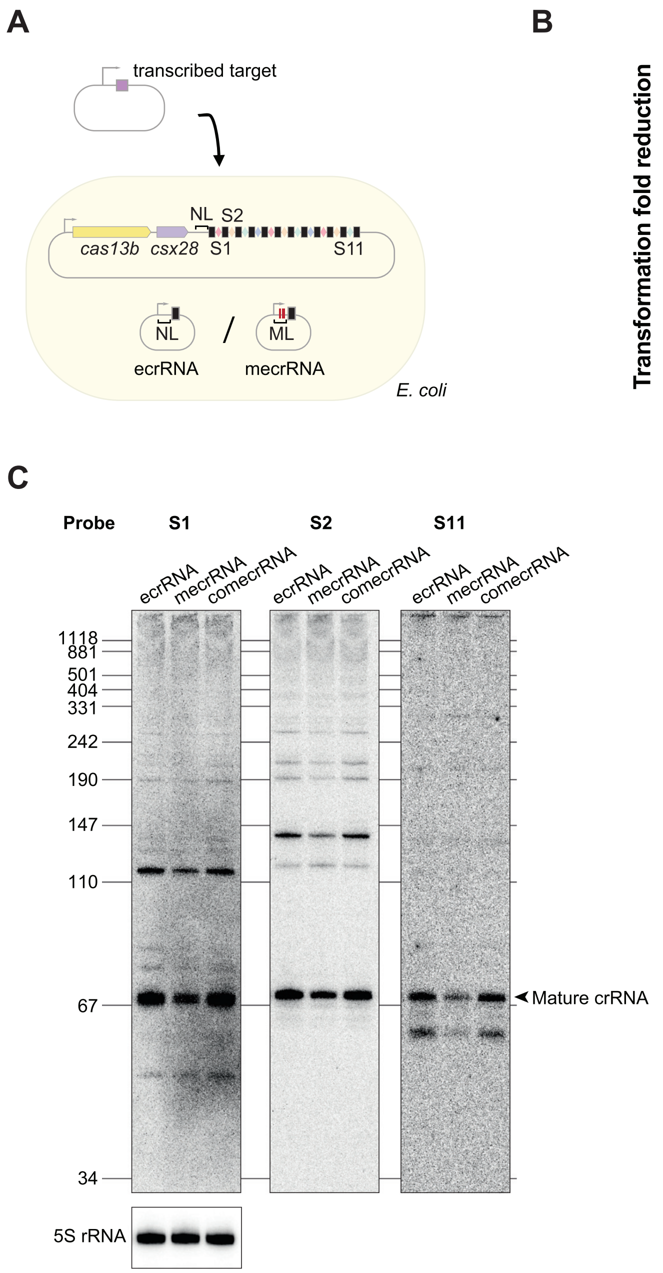

Figure EV4. Expression of the ecrRNA with the disrupted leader-repeat stem-loop in *trans* decreases crRNA levels from the Type VI-B array.

(**A**) The *E. coli* strain heterologously expressing the VI-B system from *P. gingivalis* AJW4 was co-transformed with the plasmids expressing a native ecrRNA (ecrRNA), the ecrRNA with the mutation in the leader (mecrRNA), or the ecrRNA with the mutations in the leader and repeat (comecrRNA). For the plasmid interference assay, an ampicillin-resistance plasmid expressing targets corresponding to spacers 1, 2, or 11 of the array, or a no-target control, was co-transformed with a kanamycin-resistance plasmid encoding the VI-B2 system from *P. gingivalis* AJW4 and a chloramphenicol-resistant plasmid expressing either ecrRNA or mecrRNA. The transformation was plated on triple-antibiotic selection plates. (**B**) Plasmid interference by Cas13b when expressing the ecrRNA in *trans*. The plasmid expressed a transcript targeted by spacer 1, 2, or 11, while the ecrRNA or mecrRNA was expressed in *trans*. The *p* values were calculated using a paired *t*- test with a two-tailed *p* value. The experiment was performed in two or three biological replicates. Each dot represents an independent biological replicate, while the bars and error bars represent the geometric mean and geometric standard deviation. (**C**) Northern blotting analysis of the total RNA extracted from the *E. coli* strains heterologously co-expressing the VI-B system from *P. gingivalis* AJW4 together with ecrRNA, mecrRNA, or comecrRNA. RNA was separated on 8% polyacrylamide gel with 7 M urea; ^{32}P-labeled probes to the spacers 1, 2, and 11 were hybridized. To control the RNA loading, hybridization of the probe to 5S rRNA was performed. One representative replicate out of three is shown. (**D**) Quantification of the mature crRNAs abundance levels. The bands running at the size of mature crRNAs (at 66 nts) were used to measure intensities. 5S rRNA was used to normalize the intensities. Error bars depict the standard deviation of biological triplicates. Expression of mecrRNA significantly dropped the crRNAs transcript levels compared to the control strain expressing ecrRNA for spacers 1 (2.2-fold), 2 (1.9-fold), and 11 (2.2-fold). The *p* values were calculated using a paired *t*-test with two tails. Each dot represents an independent biological replicate, while the bars and error bars represent the mean and standard deviation.

© The Author(s)

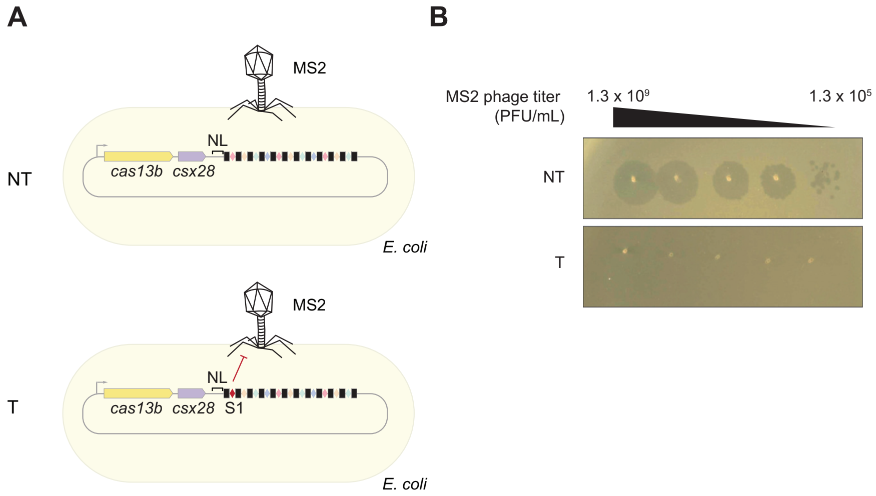

Figure EV5. The CRISPR-Cas13b system confers efficient protection against the MS2 phage.

(**A**) *E. coli* strains heterologously expressing the type VI-B CRISPR-Cas system from *P. gingivalis* AJW4, carrying either the native array (NT) or an array in which spacer 1 was replaced with an MS2-targeting spacer (T), were challenged with MS2 phage. (**B**) Plaque formation following infection with the lytic MS2 phage. A spacer targeting MS2 protected the cells from infection, resulting in the absence of visible plaques. Shown are regions of agar plates overlaid with soft agar containing *E. coli* strains, onto which serial dilutions of MS2 phage were spotted. The data were representative of three independent experiments.

 © The Author(s)
