# Supplementary material for: A leader-repeat hairpin blocks extraneous CRISPR RNA production in diverse CRISPR-Cas13 systems
Source: EMBO J. 2026 Apr 2;45(10):3396–415. doi: 10.1038/s44318-026-00769-1 (PMC13187072; doi:10.1038/s44318-026-00769-1)
Supplement: Supplementary file 6 — Peer Review File [file 44318_2026_769_MOESM6_ESM.pdf]

THE EMBO JOURNAL

A leader-repeat hairpin blocks extraneous CRISPR RNA production in diverse CRISPR-Cas13 systems

Angela Migur, Maximilian Feussner, Chunyu Liao, Omer Alkhnbashi, Adrien Chauvier, Nils Walter, Rolf Backofen, Zasha Weinberg, and Chase Beisel

Corresponding author(s): Chase Beisel (chase.beisel@helmholtz-hiri.de)

Review Timeline:

Submission Date:	25th Jun 25
Editorial Decision:	14th Aug 25
Revision Received:	16th Jan 26
Editorial Decision:	26th Feb 26
Revision Received:	28th Feb 26
Accepted:	11th Mar 26

Editor: Cornelius Schneider

Transaction Report:

(Note: With the exception of the correction of typographical or spelling errors that could be a source of ambiguity, letters and reports are not edited. Depending on transfer agreements, referee reports obtained elsewhere may or may not be included in this compilation. Referee reports are anonymous unless the Referee chooses to sign their reports.)

1st Editorial Decision 14th Aug 2025

Dear Prof. Beisel,

Thank you for submitting your manuscript for consideration by the EMBO Journal. It has now been seen by three referees whose comments are shown below.

Given the referees' positive recommendations, I would like to invite you to submit a revised version of the manuscript, addressing the comments of all three reviewers. I should add that it is EMBO Journal policy to allow only a single round of revision, and acceptance of your manuscript will therefore depend on the completeness of your responses in this revised version.

When preparing your letter of response to the referees' comments, please bear in mind that this will form part of the Review Process File, and will therefore be available online to the community. For more details on our Transparent Editorial Process, please visit our website: https://www.embopress.org/page/journal/14602075/authorguide#transparentprocess

We generally allow three months as standard revision time. As a matter of policy, competing manuscripts published during this period will not negatively impact on our assessment of the conceptual advance presented by your study. However, we request that you contact the editor as soon as possible upon publication of any related work, to discuss how to proceed. Should you foresee a problem in meeting this three-month deadline, please let us know in advance and we may be able to grant an extension.

Thank you for the opportunity to consider your work for publication. I look forward to your revision.

Yours sincerely,

Cornelius Schneider, PhD
Editor
The EMBO Journal
c.schneider@embojournal.org

Instructions for preparing your revised manuscript:

Please make sure you upload a letter of response to the referees' comments together with the revised manuscript.

Please also check that the title and abstract of the manuscript are brief, yet explicit, even to non-specialists.

When assembling figures, please refer to our figure preparation guideline in order to ensure proper formatting and readability in print as well as on screen:
https://bit.ly/EMBOPressFigurePreparationGuideline
See also guidelines for figure legends: https://www.embopress.org/page/journal/14602075/authorguide#figureformat

At EMBO Press we ask authors to provide source data for the main manuscript figures. You will receive a separate email with instructions for providing source data with your revised manuscript, including how to upload and organize the files.

IMPORTANT: When you send the revision we will require
- a point-by-point response to the referees' comments, with a detailed description of the changes made (as a word file).
- a word file of the manuscript text.
- individual production quality figure files (one file per figure)
- a complete author checklist, which you can download from our author guidelines (https://www.embopress.org/page/journal/14602075/authorguide).
- Expanded View files (replacing Supplementary Information)
Please see out instructions to authors
https://www.embopress.org/page/journal/14602075/authorguide#expandedview
- a Reagents and Tools Table as part of the Methods section, which can be downloaded from our author guidelines (https://www.embopress.org/page/journal/14602075/authorguide#structuredmethods)

Please remember: Digital image enhancement is acceptable practice, as long as it accurately represents the original data and conforms to community standards. If a figure has been subjected to significant electronic manipulation, this must be noted in the figure legend or in the 'Materials and Methods' section. The editors reserve the right to request original versions of figures and

the original images that were used to assemble the figure.

Further information is available in our Guide For Authors: https://www.embopress.org/page/journal/14602075/authorguide

We realize that it is difficult to revise to a specific deadline. In the interest of protecting the conceptual advance provided by the work, we recommend a revision within 3 months (12th Nov 2025). Please discuss the revision progress ahead of this time with the editor if you require more time to complete the revisions. Use the link below to submit your revision:

https://emboj.msubmit.net/cgi-bin/main.plex

Referee #1:

Angela et al. showed that in RNA-targeting type VI CRISPR-Cas systems, the leader RNA encoded upstream of the CRISPR array prevents formation of a seemingly useless extraneous crRNA (ecrRNA) by blocking processing of the first repeat. The work is interesting and should inform the design of Cas13-based tools, yet its physiological relevance remains unresolved (see below).

Major points:
1.The authors acknowledged that the experimental setting (expression from multi-copy plasmid, plasmid instead of phage interference was measured, etc) may hindered them from observing effects of ecrRNAs on the immunity. I suggest them to further verify these effects by refining these parameters. This is important to support the physiological relevance of the leader-repeat hairpin structure.
2.Figure 1: I found the classification of "guide-repeat" and "repeat-guide" to be likely problematic. Class 1, e.g., type I, theoretically can also produce a leader-repeat product composing of the transcribed leader sequence and a > 20 nt 3' handle derived from the first repeat (which is bound and protected by Cas6/Cas5c).

Minor points:
1.Figure 4C: the size marker need be provided. 4B: how the p value was calculated need be clarified.
2.Figure 6B: the legend need revision. "the targets were transformed?"
3.Line124: more explanation of the in-line probing assay is required here.
4.Line155: change double mutation to complementary mutation.
5.Figure 5B: 5S rRNA with S in italic.
6.Figure 6: statistical analyses are needed.
7.Table S seems to not well formatted.

Referee #2:

SUMMARY
In this article, the authors describe the function of the leader repeat in blocking the formation of extraneous crRNA (ecrRNA) by inhibiting processing of the first repeat. This study primarily focuses on type VI-B and VI-B related systems, such as VI-Bt, VI-E, VI-F, VI-G, VI-H, VI-I, VI-J that have a guide-repeat CRISPR array orientation, where the leader repeat is adjacent to the newest spacer. A comprehensive analysis of the complementarity between the leader and the first repeat in these type VI systems, showed significant base pairing between them. Structural predictions and in-line probing further confirmed this stem-loop formation between the leader and the first repeat sequence. Binding and processing of the pre-crRNA by PgiCas13b was inhibited by this leader repeat hairpin and disrupting the hairpin through mutations, caused the pre-crRNA to have higher binding affinity and processivity compared to the native locus. When expressed in E.coli, the authors were able to show through northern blots, that mature ecrRNA was only present when the leader-repeat hairpin was mutated. Mutating the leader also reduced transformation efficiency in a plasmid interference assay by 105-fold compared to the non-targeting control. Finally, disrupting the hairpin found to have no effect on the abundance of the spacers in the array or their ability to interfere with targeting plasmids, but an overall reduction of mature crRNA suggested competition between the ecrRNA and other crRNA.

Overall, the study is well executed, the data clearly presented, and the narrative concise. Except for some inconsistencies in Fig. 6, the manuscript is highly recommended for publication.

MAJOR COMMENTS
1) Figure 6C and 6D are confusing. According to the authors, the amount of crRNA reduces when ecrRNA is expressed in trans - however I only see thicker bands for all three spacers on the northern blot. Could the authors explain how they concluded that "there was a significant, ~2-fold reduction in all detected mature crRNAs (from S1, S2, S11) across the CRISPR array (Fig. 6C-D)."?

2) Based on Fig 7 and the absence of any reduction in plasmid interference presented in Figure 6A-B, the authors could test any changes to fitness with phage infection. Phage inhibition assays can be set-up by designing spacers to target one or many phages. The ecrRNA can be expressed in trans and its ability to hamper effective phage inhibition by Cas13b can be quantified as plaque forming units/mL in the presence and absence of the ecrRNA. This experiment would further strengthen the findings of this study.

MINOR COMMENTS
1) line 167- The lack of detectable ecrRNAs could be attributed to the lack of binding by PgiCas13b that would otherwise protect the ecrRNA from cellular RNases. Add "otherwise"

2) line 537, 552, 638, 659- Does the author mean 8% "PAGE", instead of "PAAG" and "PAA". This change should also be made to some of the supplementary figure legends.

3) line 573-"The black arrows indicate the newest spacer" There seem to be no spacers in figure 1A that indicate to the newest spacer.

4) Line 618- Remove "Note that the mutations to the first repeat are also expected to disrupt recognition by Cas13b"

Referee #3:

CRISPR-Cas systems use arrays of repeats and spacers to store sequences corresponding to foreign phage and plasmid sequences, which are transcribed into a pre-crRNA and processed into small, mature crRNAs that guide Cas nucleases for sequence specific nucleic acid recognition. Processing of crRNAs is mediated by sequences and secondary structures present in the pre-crRNA repeats. As CRISPR arrays are flanked by repeats on both sides, this poses a problem: one end of the pre-crRNA has the potential to form an extraneous crRNA (ecrRNA) that would occupy the Cas nuclease but does not encode a foreign sequence. This is an important problem because it could lead to autoimmunity or diminish the efficacy of limited Cas nucleases. Migur et al investigate the mechanism by which ecrRNA formation is prevented in RNA-targeting type VI CRISPR systems.

The authors find that the majority of type VI CRISPR systems form an RNA hairpin between the leader sequence upstream of the CRISPR array, and the first repeat. They perform elegant RNase footprinting experiments demonstrating that this hairpin indeed forms in the CRISPR system associated with PgiCas13b, and prevents processing of ecrRNAs, which otherwise function as inappropriate crRNAs and occupy PgiCas13b at the expense of bona fide crRNAs. Overall this is a noteworthy finding supported by well-executed experiments, and the manuscript is well-written. The only weakness in the paper is that the work is conducted in E. coli rather than the native host, however the in vitro experiments argue that hairpin formation is inherent to the pre-crRNA sequence and host-independent, and working in the native host is likely intractable. I have no other critiques and support publication.

EMBOJ-2025-121651

We are grateful to the editor and the three reviewers for their thoughtful and constructive feedback and suggestions. The reviewers were positive about our discovery that a prevalent branch of CRISPR-Cas systems suppresses ecrRNA formation, and they agreed that our data largely support our claims. Some concerns were also raised, with requests for more context or experiments to support specific claims.

Apart from revisions throughout the text, we performed a new experiment to explore whether the ecrRNA interferes with CRISPR-Cas13b immunity. The results, incorporated as Fig. 6, show that overexpressing the ecrRNA in *trans* could significantly reduce defense against the MS2 phage, supporting a selective pressure to inhibit its production as part of CRISPR evolution. The results of this experiment and the reviewers' feedback significantly strengthened our claims and provided a clearer understanding of the biological relevance of preventing an ecrRNA formation.

Specific responses to each reviewer's comments are found below, marked in blue. Corresponding changes in the main text and the supplementary information are marked in red.

Note that, during the revision process, the first author and the corresponding author changed institutions before completing additional work, leading to an update to the affiliations and the correspondence email address.

Referee #1:

Angela et al. showed that in RNA-targeting type VI CRISPR-Cas systems, the leader RNA encoded upstream of the CRISPR array prevents formation of a seemingly useless extraneous crRNA (ecrRNA) by blocking processing of the first repeat. The work is interesting and should inform the design of Cas13-based tools, yet its physiological relevance remains unresolved (see below).

We thank the reviewer for their positive comments and feedback on our efforts to resolve the biological relevance of preventing ecrRNA formation. Based on the reviewer's suggestions, we have carefully reviewed these claims and performed the suggested follow-on experiment.

Major points:

1.The authors acknowledged that the experimental setting (expression from multi-copy plasmid, plasmid instead of phage interference was measured, etc) may hindered them from observing effects of ecrRNAs on the immunity. I suggest them to further verify these effects by refining these parameters. This is important to support the physiological relevance of the leader-repeat hairpin structure.

We agree that our work would be stronger with experimental evidence supporting the physiological relevance of the leader-repeat hairpin structure. Reviewer #2 raised a similar concern and suggested a follow-on experiment that we conducted. Specifically, we performed a phage inhibition assay in which the host *E. coli* expresses a CRISPR array with one spacer targeting the MS2 phage from one plasmid and expresses the ecrRNA (i.e., with the native leader that blocks ecrRNA formation) or mecrRNA (i.e., with the mutated leader that no longer blocks ecrRNA formation) from another plasmid. As shown below, we obtained ~1,000-fold more plaques expressing the mecRNA than the ecrRNA, demonstrating that ecrRNA formation can interfere with anti-phage defense by Cas13b. This result further strengthens the physiological relevance of blocking production of extraneous crRNA.

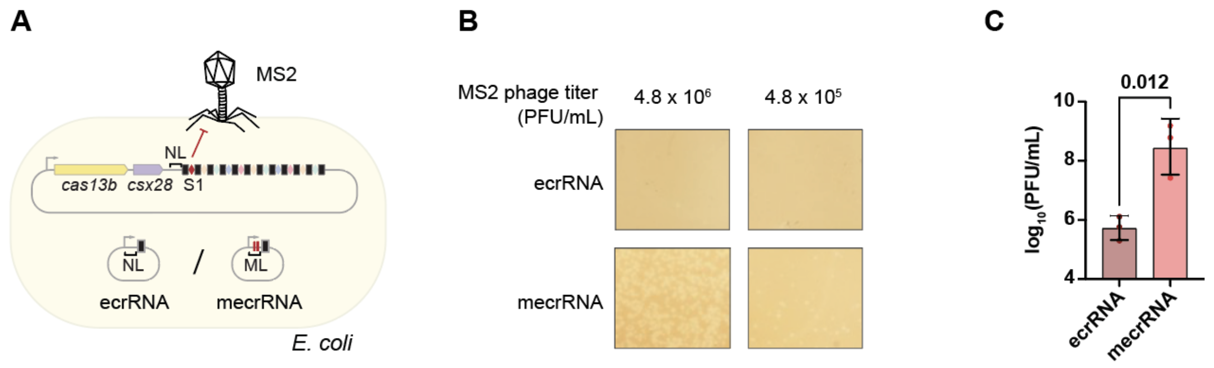

The new data were incorporated as Fig. 6, while the previous Fig. 6 now appears as Fig. S7. We also added the following to p. 10:

> "Building on this finding, we assessed the impact of ecrRNA expression on anti-phage defense. Here, we replaced spacer 1 in the native array with an MS2-targeting spacer, which conferred protection against MS2 infection in *E. coli* (**Fig. S8**). We also placed the ecrRNA on a higher-copy plasmid to drive a potentially stronger inhibitory effect. In line with this expectation, overexpression of the functional ecrRNA resulted in an ~1,000-fold increase in plaque formation compared to the non-functional ecrRNA (**Fig. 6**)."

2.Figure 1: I found the classification of "guide-repeat" and "repeat-guide" to be likely problematic. Class 1, e.g., type I, theoretically can also produce a leader-repeat product composing of the transcribed leader sequence and a > 20 nt 3' handle derived from the first repeat (which is bound and protected by Cas6/Cas5c).

We thank the reviewer for raising this point, as classifying Class 1 systems required some thought. The long repeat-derived 3′ hairpin in Class 1 CRISPR arrays might lend them to being categorized as leader–repeats. However, the 5′ handle is the "business end" that defines the crRNA, as this end is bound by Cas5 and seeds assembly of the other subunits to complete formation of the Cascade complex. In contrast, the 3′ hairpin is only necessary to process the pre-crRNAs into

individual crRNAs and free the 5′ handle for recognition by Cas5. Accordingly, loss of the 5′ handle generally abolishes function of the crRNA (10.1101/gad.1742908), while the 3′ hairpin is often dispensable or variably trimmed (10.1074/jbc.M111.238485, 10.1126/science.1159689, 10.1016/j.cell.2009.07.040). Given these points, we hold that the "leader-repeat/repeat-leader" framework is more appropriate for Class 1 CRISPR arrays, resulting in a functional ecrRNA being derived from the terminal repeat.

To provide more explanation for selecting the "leader-repeat" configuration and the ecrRNA appearing after the oldest spacer for all Class 1 systems, we added the following to the legend of Figure 1:

> "For Class I systems, the ecrRNA appears at the end since the 5′ end of each crRNA seeds effector complex formation while the 3′ end is dispensable following processing."

Minor points:
1.Figure 4C: the size marker need be provided. 4B: how the p value was calculated need be clarified.

We thank the reviewer for noting these elements. In Figure 4C, the assay was designed to monitor whether PgiCas13b processes the leader-repeat hairpin (a "yes/no" readout), so a size marker was not included. As the previously indicated lengths of the cleavage products implied size markers were present, we removed these markers.

Concerning the p-value, we added the following in the legend of Figure 4B:

> "The p value comparing the binding affinities of NL and ML was calculated using an unpaired t test with a two-tailed p value."

2.Figure 6B: the legend need revision. "the targets were transformed?"

We thank the reviewer for pointing out the unclear wording in the legend. We have revised the corresponding legend now part of Figure S7 to clarify the experimental setup with the following:

> "For the plasmid interference assay, an ampicillin-resistance plasmid expressing targets corresponding to spacers 1, 2, or 11 of the array, or a no-target control was co-transformed with a kanamycin-resistance plasmid encoding the VI-B2 system from *P. gingivalis* AJW4 and a chloramphenicol-resistant plasmid expressing either ecrRNA or mecrRNA."

3.Line124: more explanation of the in-line probing assay is required here.

We thank the reviewer for the suggestion. As requested, we added the following on p. 7:

"To resolve the secondary structure of the leader–repeat RNA, we performed in-line probing that reveals single-stranded regions of folded RNAs *in vitro* based on their propensity to undergo self-cleavage through nucleophilic attack of the phosphate backbone driven by the adjacent ribose hydroxyl group[22]."

4.Line155: change double mutation to complementary mutation.

Following the reviewer's suggestion, we phrased the sentence on p. 8 to read the following:

"Introducing compensatory mutations in the repeat resulted in undetectable processing."

Here, we felt "compensatory" is more appropriate since it's restoring the base-pairing interaction.

5.Figure 5B: 5S rRNA with S in italic.

The intent was to specify the detection of 5S rRNA. We have now written "5S rRNA" instead of "5S".

6.Figure 6: statistical analyses are needed.

Following the reviewer's suggestion, we incorporated statistical analyses into the figure (now Fig. S7). The analyses are now noted in the legend.

7.Table S seems to not well formatted.

As suggested by the reviewer, we revisited the .xlsx file and updated the formatting where needed to improve readability.

Referee #2:

SUMMARY

In this article, the authors describe the function of the leader repeat in blocking the formation of extraneous crRNA (ecrRNA) by inhibiting processing of the first repeat. This study primarily focuses on type VI-B and VI-B related systems, such as VI-Bt, VI-E, VI-F, VI-G, VI-H, VI-I, VI-J that have a guide-repeat CRISPR array orientation, where the leader repeat is adjacent to the newest spacer. A comprehensive analysis of the complementarity between the leader and the first repeat in these type VI systems, showed significant base pairing between them. Structural predictions and in-line probing further confirmed this stem-loop formation between the leader and the first repeat sequence. Binding and processing of the pre-crRNA by PgiCas13b was inhibited by this leader repeat hairpin and disrupting the hairpin through mutations, caused the pre-crRNA to have higher binding affinity and processivity compared to the native locus. When expressed in E.coli, the authors were able to show through northern blots, that mature ecrRNA was only present when the leader-repeat hairpin was mutated. Mutating the leader also reduced transformation efficiency in a plasmid interference assay by 105-fold compared to the non-targeting control. Finally, disrupting the hairpin found to have no effect on the abundance of the spacers in the array or their ability to interfere with targeting plasmids, but an overall reduction of mature crRNA suggested competition between the ecrRNA and other crRNA.

Overall, the study is well executed, the data clearly presented, and the narrative concise. Except for some inconsistencies in Fig. 6, the manuscript is highly recommended for publication.

We thank the reviewer for their supportive and helpful comments. We addressed each of the raised issues below with an additional experiment paralleling the one requested by Reviewer #1 (see Major Comment #1). We detail our responses to each of the reviewer's concerns below.

MAJOR COMMENTS

1) Figure 6C and 6D are confusing. According to the authors, the amount of crRNA reduces when ecrRNA is expressed in trans - however I only see thicker bands for all three spacers on the northern blot. Could the authors explain how they concluded that "there was a significant, ~2-fold reduction in all detected mature crRNAs (from S1, S2, S11) across the CRISPR array (Fig. 6C-D)."?

This conclusion came from quantifying the band intensities across replicates at the expected size of the mature crRNA (~70 nts). For each of the spacers, expressing the mecrRNA (i.e., the version that forms a functional ecrRNA) resulted in lower mature crRNA levels than those when expressing the ecrRNA and the comecrRNA (i.e., versions that do not form a functional crRNA). The changes were slight but reproducible and statistically significant. These results are captured in the following on p. 10:

"However, there was a small but significant reduction in all detected mature crRNAs (from S1, S2, S11) across the CRISPR array (**Fig. S7C-D**), suggesting some effect of functional ecrRNA expression in *trans*."

This reduction is also now less important for the storyline with the inclusion of phage defense (Fig. 6).

2) Based on Fig 7 and the absence of any reduction in plasmid interference presented in Figure 6A-B, the authors could test any changes to fitness with phage infection. Phage inhibition assays can be set-up by designing spacers to target one or many phages. The ecrRNA can be expressed in trans and its ability to hamper effective phage inhibition by Cas13b can be quantified as plaque forming units/mL in the presence and absence of the ecrRNA. This experiment would further strengthen the findings of this study.

The reviewer makes an excellent suggestion that parallels the comment made by Reviewer #1 (Major Comment #1). Closely following this Reviewer #2's suggestion, we designed a spacer targeting the MS2 phage and replaced spacer 1 in the native array with this MS2-targeting spacer. This spacer confers protection to *E. coli* cells against MS2 infection.

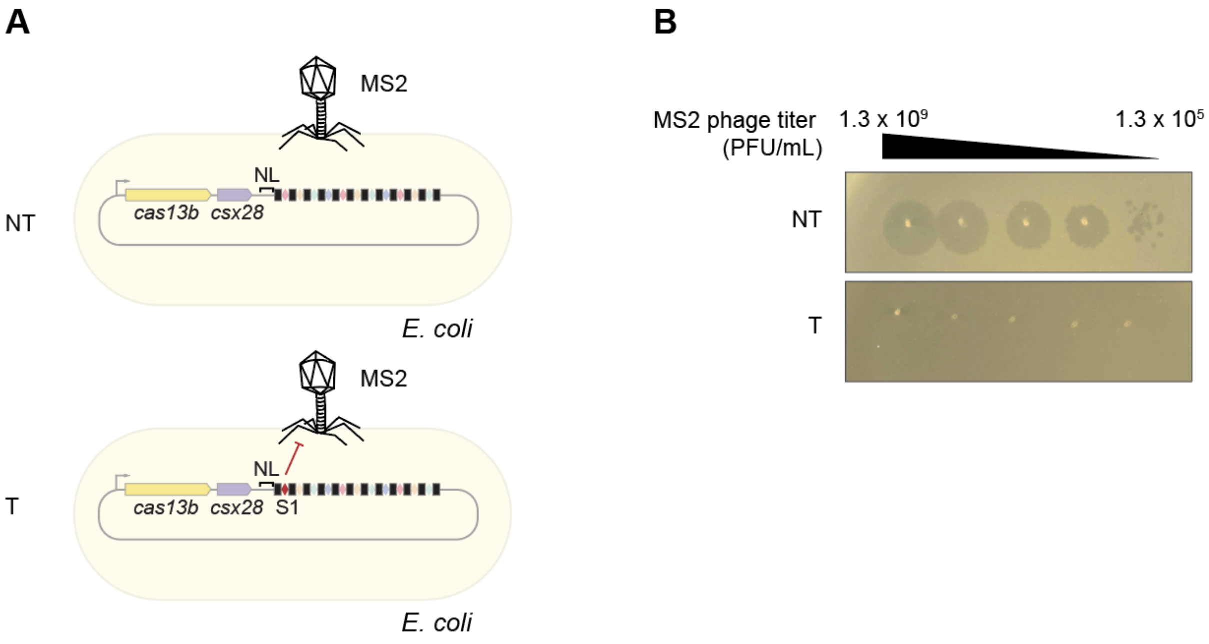

These new data were incorporated as Fig. S8.

We next examined whether overexpression of the mecrRNA versus the ecrRNA in *trans* affects Cas13b-mediated phage defense. As a control, we used a strain overexpressing a non-functional ecrRNA in which the leader and repeat form a stem-loop. In contrast, we found that a functional

ecrRNA with a disrupted leader-repeat stem-loop can sequester Cas13b and thereby impair phage protection when overexpressed in *trans*.

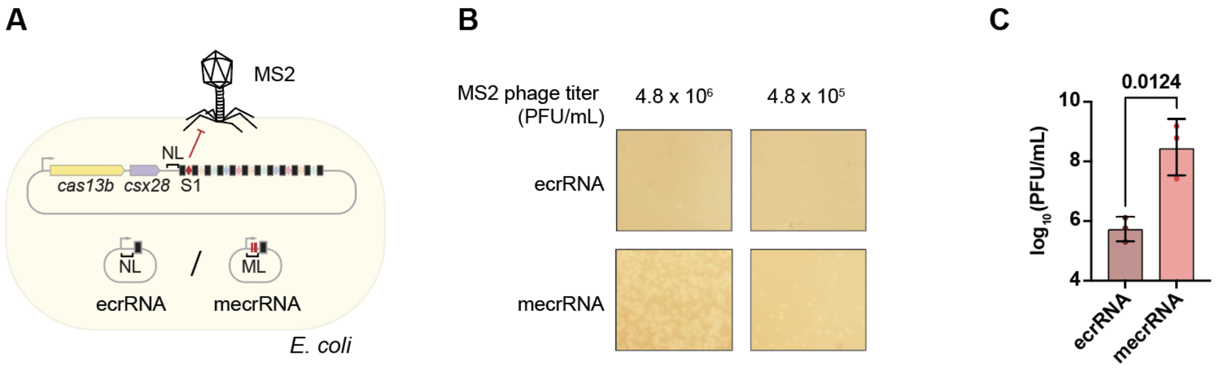

The new data were incorporated as Fig. 6, while the previous Fig. 6 now appears as Fig. S7.

We also added the following to p. 10:

> "Building on this finding, we assessed the impact of ecrRNA expression on anti-phage defense. Here, we replaced spacer 1 in the native array with an MS2-targeting spacer, which conferred protection against MS2 infection in *E. coli* (**Fig. S8**). We also placed the ecrRNA on a higher-copy plasmid to drive a potentially stronger inhibitory effect. In line with this expectation, overexpression of the functional ecrRNA resulted in an ~1,000-fold increase in plaque formation compared to the non-functional ecrRNA (**Fig. 6**)."

MINOR COMMENTS

1) line 167- The lack of detectable ecrRNAs could be attributed to the lack of binding by PgiCas13b that would otherwise protect the ecrRNA from cellular RNases. Add "otherwise"

We thank the reviewer for noting this caveat. The associated sentence on p. 9 now reads as follows:

> "The lack of detectable ecrRNAs could be attributable to the lack of binding by PgiCas13b that would otherwise protect the ecrRNA from cellular RNases."

2) line 537, 552, 638, 659- Does the author mean 8% "PAGE", instead of "PAAG" and "PAA". This change should also be made to some of the supplementary figure legends.

To improve clarity, we replaced the abbreviation PAAG with "polyacrylamide gel".

3) line 573-"The black arrows indicate the newest spacer" There seem to be no spacers in figure 1A that indicate to the newest spacer.

We thank the Reviewer for catching this error. We have removed the sentence from the legend of Figure 1A.

4) Line 618- Remove "Note that the mutations to the first repeat are also expected to disrupt recognition by Cas13b"

Following the reviewer's suggestion, we have removed this line.

Referee #3:

CRISPR-Cas systems use arrays of repeats and spacers to store sequences corresponding to foreign phage and plasmid sequences, which are transcribed into a pre-crRNA and processed into small, mature crRNAs that guide Cas nucleases for sequence specific nucleic acid recognition. Processing of crRNAs is mediated by sequences and secondary structures present in the pre-crRNA repeats. As CRISPR arrays are flanked by repeats on both sides, this poses a problem: one end of the pre-crRNA has the potential to form an extraneous crRNA (ecrRNA) that would occupy the Cas nuclease but does not encode a foreign sequence. This is an important problem because it could lead to autoimmunity or diminish the efficacy of limited Cas nucleases. Migur et al investigate the mechanism by which ecrRNA formation is prevented in RNA-targeting type VI CRISPR systems.

The authors find that the majority of type VI CRISPR systems form an RNA hairpin between the leader sequence upstream of the CRISPR array, and the first repeat. They perform elegant RNase footprinting experiments demonstrating that this hairpin indeed forms in the CRISPR system associated with PgiCas13b, and prevents processing of ecrRNAs, which otherwise function as inappropriate crRNAs and occupy PgiCas13b at the expense of bona fide crRNAs. Overall this is a noteworthy finding supported by well-executed experiments, and the manuscript is well-written. The only weakness in the paper is that the work is conducted in E. coli rather than the native host, however the in vitro experiments argue that hairpin formation is inherent to the pre-crRNA sequence and host-independent, and working in the native host is likely intractable. I have no other critiques and support publication.

We thank the reviewer for the supportive feedback. We acknowledge the concern that the experiments were performed in *E. coli* rather than in the native host. Many CRISPR–Cas systems are currently identified through metagenomic sequencing or originate from bacteria that are not readily culturable. In such cases, the native strains are often unavailable or cannot be cultured under laboratory conditions. Moreover, universal genetic tools for manipulation of diverse bacterial hosts and knowledge of their cognate phage invaders are frequently lacking. Therefore, we performed the experiments either *in vitro* or in *E. coli*, for which robust genetic tools are broadly available, enabling controlled and reproducible functional analyses.

To also specify the limitation of solely working in a heterologous system, we added the following to the Discussion on p. 12:

> "Expressing the processed and mature functional ecrRNA from the VI-B2 system in *trans* indicated sequestering of available Cas13b nuclease molecules that reduced Cas13b-mediated phage defense by ~1,000-fold (**Fig. 6**). This reduction required overexpression of the functional ecrRNA in *trans* that would likely be less pronounced in a native system in which only a single ecrRNA is expressed at the beginning of the array. However, crRNAs from the beginning of an array are the most highly expressed, which could introduce sufficient selective pressure to drive silencing of the ecrRNA through formation of the leader-repeat stem-loop. The selective pressure could be enhanced by longer

CRISPR arrays or in the presence of Acr-expressing phages[28,29] in which immunity becomes sensitive to perturbations in the levels of individual crRNAs."

Dear Prof. Beisel,

Thank you for submitting a revised version of your manuscript. Your study has now been seen by all original referees, who find that their previous concerns have been addressed and now recommend publication of the manuscript. There remain only a few mainly editorial points that have to be addressed before I can extend formal acceptance of the manuscript:

- Please provide an up-to-date email address for co-author Angela Migur, so that acknowledgement emails from our office can be delivered to them.

- FUNDING: the National Science Foundation (MCB 2140320 to N.G.W.) is misisng in eJP

- As we are switching from a free-text author contribution statement towards a more formal statement based on Contributor Role Taxonomy (CRediT) terms, please remove the present Author Contribution section and instead specify each author's contribution(s) directly in the Author Information page of our submission system during upload of the final manuscript. See https://casrai.org/credit/ for more information.

- Please adjust the format of the reference list and of the in-text citations according to EMBO Journal format (alphabetical order, author name et al + year.../up to 10 author names in the reference list before et al / please refer to our Guide to Authors for additional information on EMBO J reference format).

- DATASET EV LEGENDS: Table EV1 and EV2 should be renamed Dataset EV1 and EV2 and the remaining EV table files should be renumbered accordingly; all files need legends removed from the manuscript text and added to the corresponding excel files

- APPENDIX 1 FILE WITH ToC: please add page numbers to the table of contents and remove red font

- SOURCE DATA: There is a discrepancy in Figure 2 (not checked in the list, but 2A provided); The Source data also need to be uploaded as 1 folder per figure

- Please upload the REAGENT TABLE as a separate file, citations for tables will need to be updated

- Please provide suggestions for a short 'blurb' text prefacing and summing up the conceptual aspect of the study in two sentences (max. 250 characters), followed by 3-5 one-sentence 'bullet points' with brief factual statements of key results of the paper; they will form the basis of an editor-written 'Synopsis' accompanying the online version of the article. Please also provide an altered synopsis image, making sure that the aspect ratio conforms to our website's format - it should be exactly 550 pixels wide and between 300-600 pixels high.

- Please adjust the in-text callouts for individual figures and figure panels: e.g. some of the panels in Fig 2 and 5 appear to be missing, pls check and correct the order of the EV tables so that they are cited sequentially

Figure Legends:

- Please note that information related to n is missing in the legend of figure 6C

- Please note that the measure of center for the error bars needs to be defined in the legend of figure 4B"

Please let me know if you have any questions regarding any of these points. You can use the link below to upload the revised files.
Thank you again for giving us the chance to consider your manuscript for The EMBO Journal. I look forward to receiving the final version.

With best regards,

Cornelius Schneider

Cornelius Schneider, PhD
Editor | The EMBO Journal
c.schneider@embojournal.org

Please refer to our figure preparation guideline in order to ensure proper formatting and readability in print as well as on screen:

https://link.springer.com/journal/44318/submission-guidelines#cms-Figure-and-data-presentation

Use the link below to submit your revision:

https://emboj.msubmit.net/cgi-bin/main.plex

Referee #1:

The authors have made considerable efforts to revise the manuscript, including the addition of new experiments and an appropriately expanded discussion. The data presented in new Fig. 6 are convincing and strengthen the conclusions.

Regarding Fig. 1A, I still recommend adding a red asterisk to the last repeat of Class 1 to highlight that the degeneration of this repeat prevents extensive ecrRNA production. This point should be explained concisely in the figure legend itself, rather than relying solely on the main text, to avoid potential misinterpretation by readers.

Aside from this minor suggestion, the authors have satisfactorily addressed all of my concerns.

Referee #2:

The authors have done a great job addressing the comments by providing clear phage infection data that support their model for the extraneous CRISPR RNA. With the inclusion of these data, I have no further comments and recommend for publication.

Referee #3:

I had essentially no critiques for the authors to address, and they also did an excellent job addressing the concerns of the other reviewers, which were very minor. I recommend publication.

EMBOJ-2025-121651

We thank the reviewers for their positive feedback on our revised manuscript. Responses to the remaining comments are below.

Referee #1:

The authors have made considerable efforts to revise the manuscript, including the addition of new experiments and an appropriately expanded discussion. The data presented in new Fig. 6 are convincing and strengthen the conclusions.

Regarding Fig. 1A, I still recommend adding a red asterisk to the last repeat of Class 1 to highlight that the degeneration of this repeat prevents extensive ecrRNA production. This point should be explained concisely in the figure legend itself, rather than relying solely on the main text, to avoid potential misinterpretation by readers.

Aside from this minor suggestion, the authors have satisfactorily addressed all of my concerns.

We thank the reviewer for the follow-on suggestion to provide more clarity around ecrRNAs derived from Class 1 systems. Following the reviewer's suggestion, we added a red asterisk to "Class 1", with a callout to this punctuation in the legend. The red asterisk was also added for II-B systems since a parallel explanation is provided in the same legend.

Referee #2:

The authors have done a great job addressing the comments by providing clear phage infection data that support their model for the extraneous CRISPR RNA. With the inclusion of these data, I have no further comments and recommend for publication.

Referee #3:

I had essentially no critiques for the authors to address, and they also did an excellent job addressing the concerns of the other reviewers, which were very minor. I recommend publication.

Dear Prof. Beisel,

I am pleased to inform you that your manuscript has been accepted for publication in the EMBO Journal.

Your manuscript will be processed for publication by EMBO Press. It will be copy edited and you will receive page proofs prior to publication. Please note that you will be contacted by Springer Nature Author Services to complete licensing and payment information.

You may qualify for financial assistance for your publication charges - either via a Springer Nature fully open access agreement or an EMBO initiative. Check your eligibility: https://link.springer.com/journal/44318/how-to-publish-with-us

Should you be planning a Press Release on your article, please get in contact with embo_production@springernature.com as early as possible in order to coordinate publication and release dates.

If you have any questions, please do not hesitate to contact the Editorial Office. Thank you for your contribution to The EMBO Journal.

Yours sincerely,

Cornelius Schneider, PhD
Editor
The EMBO Journal
c.schneider@embojournal.org

*****
Please note that it is The EMBO Journal policy for the transcript of the editorial process (containing referee reports and your response letters) to be published as an online supplement to each paper. If you should prefer removal of any referee-only figures included in the point-by-point response(s), e.g. because they may still be used for future publication or because they have been reproduced from published work by others, please do let us know immediately via response email.
More information is available here: https://link.springer.com/partners/embo-press/editorial-policies#Peer%20review

** Click here to be directed to your login page: https://emboj.msubmit.net

EMBO Press Author Checklist

Corresponding Author Name: Chase L. Beisel	
Journal Submitted to: The EMBO Journal	
Manuscript Number: EMBOJ-2025-121651	

USEFUL LINKS FOR COMPLETING THIS FORM
The EMBO Journal - Author Guidelines
EMBO Reports - Author Guidelines
Molecular Systems Biology - Author Guidelines
EMBO Molecular Medicine - Author Guidelines

Reporting Checklist for Life Science Articles (updated January
This checklist is adapted from Materials Design Analysis Reporting (MDAR) Checklist for Authors. MDAR establishes a minimum set of requirements in transparent reporting in the life sciences (see Statement of Task: 10.31222/osf.io/9sm4x). Please follow the journal's guidelines in preparing your
Please note that a copy of this checklist will be published alongside your article.

Abridged guidelines for figures
1. Data
The data shown in figures should satisfy the following conditions:
- the data were obtained and processed according to the field's best practice and are presented to reflect the results of the experiments in an accurate and unbiased manner.
- ideally, figure panels should include only measurements that are directly comparable to each other and obtained with the same assay.
- plots include clearly labeled error bars for independent experiments and sample sizes. Unless justified, error bars should not be shown for technical
- if n<5, the individual data points from each experiment should be plotted. Any statistical test employed should be justified.
- Source Data should be included to report the data underlying figures according to the guidelines set out in the authorship guidelines on Data

2. Captions
Each figure caption should contain the following information, for each panel where they are relevant:
- a specification of the experimental system investigated (eg cell line, species name).
- the assay(s) and method(s) used to carry out the reported observations and measurements.
- an explicit mention of the biological and chemical entity(ies) that are being measured.
- an explicit mention of the biological and chemical entity(ies) that are altered/varied/perturbed in a controlled manner.
- the exact sample size (n) for each experimental group/condition, given as a number, not a range;
- a description of the sample collection allowing the reader to understand whether the samples represent technical or biological replicates (including how many animals, litters, cultures, etc.).
- a statement of how many times the experiment shown was independently replicated in the laboratory.
- definitions of statistical methods and measures:
  - common tests, such as t-test (please specify whether paired vs. unpaired), simple χ2 tests, Wilcoxon and Mann-Whitney tests, can be unambiguously identified by name only, but more complex techniques should be described in the methods section;
  - are tests one-sided or two-sided?
  - are there adjustments for multiple comparisons?
  - exact statistical test results, e.g., P values = x but not P values < x;
  - definition of 'center values' as median or average;
  - definition of error bars as s.d. or s.e.m.

Please complete ALL of the questions below.
Select "Not Applicable" only when the requested information is not relevant for your study.

Materials

Newly Created Materials	Information included in the manuscript?	In which section is the information available? (Reagents and Tools Table, Materials and Methods, Figures, Data Availability Section)
New materials and reagents need to be available; do any restrictions apply?	Yes	Reagents and Tools Table

Antibodies	Information included in the manuscript?	In which section is the information available? (Reagents and Tools Table, Materials and Methods, Figures, Data Availability Section)
For **antibodies** provide the following information:<br>- Commercial antibodies: RRID (if possible) or supplier name, catalogue number and or/clone number<br>- Non-commercial: RRID or citation	Not Applicable	

DNA and RNA sequences	Information included in the manuscript?	In which section is the information available? (Reagents and Tools Table, Materials and Methods, Figures, Data Availability Section)
**Short novel DNA or RNA including primers, probes**: provide the sequences.	Yes	Reagents and Tools Table

Cell materials	Information included in the manuscript?	In which section is the information available? (Reagents and Tools Table, Materials and Methods, Figures, Data Availability Section)
**Cell lines:** Provide species information, strain. Provide accession number in repository **OR** supplier name, catalog number, clone number, and/**OR** RRID.	Not Applicable	
**Primary cultures:** Provide species, strain, sex of origin, genetic modification status.	Not Applicable	
Report if the cell lines were recently **authenticated** (e.g., by STR profiling) and tested for mycoplasma contamination.	Not Applicable	

Experimental animals	Information included in the manuscript?	In which section is the information available? (Reagents and Tools Table, Materials and Methods, Figures, Data Availability Section)
**Laboratory animals or Model organisms:** Provide species, strain, sex, age, genetic modification status. Provide accession number in repository **OR** supplier name, catalog number, clone number, **OR** RRID.	Not Applicable	
**Animal observed in or captured from the field:** Provide species, sex, and age where possible.	Not Applicable	
Please detail **housing and husbandry conditions**.	Not Applicable	

Plants and microbes	Information included in the manuscript?	In which section is the information available? (Reagents and Tools Table, Materials and Methods, Figures, Data Availability Section)
**Plants:** provide species and strain, ecotype and cultivar where relevant, unique accession number if available, and source (including location for collected wild specimens).	Not Applicable	
**Microbes:** provide species and strain, unique accession number if available, and source.	Yes	Reagents and Tools Table

Human research participants	Information included in the manuscript?	In which section is the information available? (Reagents and Tools Table, Materials and Methods, Figures, Data Availability Section)
If collected and within the bounds of privacy constraints report on age, sex and gender or ethnicity for all study participants.	Not Applicable	

Core facilities	Information included in the manuscript?	In which section is the information available? (Reagents and Tools Table, Materials and Methods, Figures, Data Availability Section)
If your work benefited from core facilities, was their service mentioned in the acknowledgments section?	Yes	Acknowledgments

Design

Study protocol	Information included in the manuscript?	In which section is the information available? (Reagents and Tools Table, Materials and Methods, Figures, Data Availability Section)
If study protocol has been **pre-registered, provide DOI in the manuscript**. For clinical trials, provide the trial registration number **OR** cite DOI.	Not Applicable	
Report the **clinical trial registration number** (at ClinicalTrials.gov or equivalent), where applicable.	Not Applicable	

Laboratory protocol	Information included in the manuscript?	In which section is the information available? (Reagents and Tools Table, Materials and Methods, Figures, Data Availability Section)
Provide DOI OR other citation details if **external detailed step-by-step protocols** are available.	Not Applicable	

Experimental study design and statistics	Information included in the manuscript?	In which section is the information available? (Reagents and Tools Table, Materials and Methods, Figures, Data Availability Section)
Include a statement about **sample size** estimate even if no statistical methods were used.	Yes	Materials and Methods, Figures
Were any steps taken to minimize the effects of subjective bias when allocating animals/samples to treatment (e.g. **randomization procedure**)? If yes, have they been described?	Not Applicable	
Include a statement about **blinding** even if no blinding was done.	Not Applicable	
Describe **inclusion/exclusion criteria** if samples or animals were excluded from the analysis. Were the criteria pre-established?<br><br>If sample or data points were omitted from analysis, report if this was due to attrition or intentional exclusion and provide justification.	Not Applicable	
For every figure, are **statistical tests** justified as appropriate? Do the data meet the assumptions of the tests (e.g., normal distribution)? Describe any methods used to assess it. Is there an estimate of variation within each group of data? Is the variance similar between the groups that are being statistically compared?	Yes	Materials and Methods, Figures

Sample definition and in-laboratory replication	Information included in the manuscript?	In which section is the information available? (Reagents and Tools Table, Materials and Methods, Figures, Data Availability Section)
In the figure legends: state number of times the experiment was **replicated** in laboratory.	Yes	Figures
In the figure legends: define whether data describe **technical or biological replicates**.	Yes	Figures

Ethics

Ethics	Information included in the manuscript?	In which section is the information available? (Reagents and Tools Table, Materials and Methods, Figures, Data Availability Section)
Studies involving **human participants**: State details of **authority granting ethics approval** (IRB or equivalent committee(s), provide reference number for approval.	Not Applicable	
Studies involving **human participants**: Include a statement confirming that **informed consent** was obtained from all subjects and that the experiments conformed to the principles set out in the WMA Declaration of Helsinki and the Department of Health and Human Services Belmont Report.	Not Applicable	
Studies involving **human participants:** For publication of **patient photos**, include a statement confirming that consent to publish was obtained.	Not Applicable	
Studies involving experimental **animals**: State details of **authority granting ethics approval** (IRB or equivalent committee(s), provide reference number for approval. Include a statement of compliance with ethical regulations.	Not Applicable	
Studies involving **specimen and field samples:** State if relevant **permits** obtained, provide details of authority approving study; if none were required, explain why.	Not Applicable	

Dual Use Research of Concern (DURC)	Information included in the manuscript?	In which section is the information available? (Reagents and Tools Table, Materials and Methods, Figures, Data Availability Section)
Could your study fall under dual use research restrictions? Please check biosecurity documents and list of **select agents and toxins** (CDC): https://www.selectagents.gov/sat/list.htm	Not Applicable	
If you used a select agent, is the security level of the lab appropriate and reported in the manuscript?	Not Applicable	
If a study is subject to dual use research of concern regulations, is the name of the **authority granting approval and reference number** for the regulatory approval provided in the manuscript?	Not Applicable	

Reporting

The MDAR framework recommends adoption of discipline-specific guidelines, established and endorsed through community initiatives. Journals have their own policy about requiring specific guidelines and recommendations to complement MDAR.

Adherence to community standards	Information included in the manuscript?	In which section is the information available? (Reagents and Tools Table, Materials and Methods, Figures, Data Availability Section)
State if relevant guidelines or checklists (e.g., **ICMJE, MIBBI, ARRIVE, PRISMA**) have been followed or provided.	Not Applicable	
For **tumor marker prognostic studies,** we recommend that you follow the **REMARK** reporting guidelines (see link list at top right). See author guidelines, under 'Reporting Guidelines'. Please confirm you have followed these guidelines.	Not Applicable	
For **phase II and III randomized controlled trials**, please refer to the **CONSORT** flow diagram (see link list at top right) and submit the CONSORT checklist (see link list at top right) with your submission. See author guidelines, under 'Reporting Guidelines'. Please confirm you have submitted this list.	Not Applicable	

Data Availability

Data availability	Information included in the manuscript?	In which section is the information available? (Reagents and Tools Table, Materials and Methods, Figures, Data Availability Section)
Have **primary datasets** been deposited according to the journal's guidelines (see 'Data Deposition' section) and the respective accession numbers provided in the Data Availability Section?	Not Applicable	
Were **human clinical and genomic datasets** deposited in a public access-controlled repository in accordance to ethical obligations to the patients and to the applicable consent agreement?	Not Applicable	
Are **computational models** that are central and integral to a study available without restrictions in a machine-readable form? Were the relevant accession numbers or links provided?	Not Applicable	
If publicly available data were reused, provide the respective **data citations in the reference list**.	Not Applicable	
